# Secure Distributed Detection under Energy Constraint in IoT-Oriented Sensor Networks

**DOI:** 10.3390/s16122152

**Published:** 2016-12-16

**Authors:** Guomei Zhang, Hao Sun

**Affiliations:** 1 School of Electronic and Information Engineering, Xi’an Jiaotong University, No. 28 West Xianning Road, Xi’an 710049, China; haosun@stu.xjtu.edu.cn; 2 Shaanxi Engineering Research Center of Smart Networks and Ubiquitous Access, Xi’an Jiaotong University, Xi’an 710049, China

**Keywords:** Internet of Things, wireless sensor network, distributed detection, eavesdropping, physical layer security, energy constraint, decision fusion

## Abstract

We study the secure distributed detection problems under energy constraint for IoT-oriented sensor networks. The conventional channel-aware encryption (CAE) is an efficient physical-layer secure distributed detection scheme in light of its energy efficiency, good scalability and robustness over diverse eavesdropping scenarios. However, in the CAE scheme, it remains an open problem of how to optimize the key thresholds for the estimated channel gain, which are used to determine the sensor’s reporting action. Moreover, the CAE scheme does not jointly consider the accuracy of local detection results in determining whether to stay dormant for a sensor. To solve these problems, we first analyze the error probability and derive the optimal thresholds in the CAE scheme under a specified energy constraint. These results build a convenient mathematic framework for our further innovative design. Under this framework, we propose a hybrid secure distributed detection scheme. Our proposal can satisfy the energy constraint by keeping some sensors inactive according to the local detection confidence level, which is characterized by likelihood ratio. In the meanwhile, the security is guaranteed through randomly flipping the local decisions forwarded to the fusion center based on the channel amplitude. We further optimize the key parameters of our hybrid scheme, including two local decision thresholds and one channel comparison threshold. Performance evaluation results demonstrate that our hybrid scheme outperforms the CAE under stringent energy constraints, especially in the high signal-to-noise ratio scenario, while the security is still assured.

## 1. Introduction

With the rapid advances in low-cost wireless sensors, radio frequency identification (RFID), Web technologies and wireless communications recently, connecting various smart objects to Internet and realizing the communications of machine-to-human and machine-to-machine with the physical world have been expected widely [1]. That is the concept of Internet of Things (IoT), which can provide ubiquitous connectivity, information gathering and data transmitting capabilities in different fields, such as health monitoring, emergencies, environment control, military and industries. The pervasive sensing and control capabilities brought by IoT will change our daily life significantly [2,3,4].

In an era of IoT, there are billions of devices linked to the Internet. Cisco predicts that 50 billion devices are going to be in use in 2020 [3]. Such a large number of devices deployed in the IoT lead to many technical challenges including spectrum scarcity, energy consumption and security [4,5,6]. Aiming to the spectrum scarcity problem, some enhanced technologies with high spectrum efficiency are advocated, for example, the cognitive Internet of Things (CIoT) who introduces the cognitive radio technology to the IoT network [5]. A decentralized inference network where the nodes transmit the compressed observations to reduce the required bandwidth is another solution [7], and the distributed detection technique utilized in sensor networks is a typical instance [8,9,10,11]. Since a huge number of devices are included in IoT, the energy to be spent for communication and computation is extremely large and improving energy efficiency becomes more important. Although the energy harvesting techniques can use the external energy source and relieve devices from the constraints induced by battery usage, energy as a scarce resource should always be utilized carefully. Thus, an energy efficiency solution has a significant role in IoT [4,12]. With the developing of IoT network, devices will become smarter and start to handle more tasks of human. Thus, the devices have to be more reliable and trustable [1]. However, there are a variety of attacks over different protocol layers which attempt to disrupt the network or intercept the information in the IoT, including denial of service (DoS) attacks, spoofed routing information attacks at network layer, flooding attacks at transport layer, resource exhaustion attacks at link layer, jamming and tampering attacks at physical layer and many others [13]. Now security has turned into an important aspect for IoT deployments [14,15]. Among various attacks, eavesdropping attack is the most common form of attack on data privacy [2,13]. In order to realize secure transmission, traditional key-based enciphering techniques at network layer have been entrusted. However, in IoT networks with low-complex devices, the key distribution for symmetric cryptosystems and the highly complex computation of asymmetric cryptosystems can be very challenging [16]. Therefore, the robust physical-layer security methods with little or no aid of encryption key and with low computational complexity can be adopted in IoT [2,5,17], further, they could be combined with other lightweight cryptographic protocols to fulfill different security targets of IoT.

An IoT system would integrate various technologies and communications solutions, such as identification and tracking techniques, wired and wireless sensor and actuator networks and enhanced communication protocols [1,18,19,20]. Sensor networks, especially the wireless sensor networks (WSN), will play a crucial role in the IoT. Ubiquitous sensing provided by WSN can offer the ability to measure, infer and understand environmental indicators. Cooperating with RFID system, WSN can track the status of things better and build a bridge between the physical and digital world [18,21]. With the size and complication of WSN growing, the spectrum scarcity and energy consumption problems become more serious [22]. Furthermore, the broadcasting nature of wireless communications from sensors to the controllers or fusion centers makes WSN vulnerable to eavesdropping. The physical layer security solutions with low complexity and low overhead are obviously more suitable for WSN, since the sensors have some practical constraints including limited computing capabilities, limited storage memories and severe energy constraints [2,10].

Due to the low bandwidth and power requirement at sensors and the robustness to the environments’ rapid changes, distributed detection in WSN has been utilized in a wide range of fields such as emergency response, environment monitoring, medical monitoring and military surveillance [10,23]. For distributed detection, sensors are deployed over a certain area to sense the physical phenomena with binary state in a decentralized fashion. Each sensor makes a binary decision based on its local observation and then transmits the local decision to a fusion center (FC) over wireless channels [23]. For the practical resource constraints and the serious security issues in front of WSN, secure distributed detection schemes under energy constraints are necessary for the development of an efficient IoT. Various secure strategies for distributed detection have been proposed under different assumptions on the eavesdroppers and transmission channels [8,9,10,23,24,25,26,27,28,29]. However, these studies focused on either the local detection at sensors or the information transmission from sensors to the FC. Moreover, the vast majority of them did not involve an energy constraint. Therefore, an efficient hybrid solution combining the local decision with the transmission under an energy constraint, along with a mathematic framework of analyzing error performance and optimizing parameters for the developed schemes are selected as the research contents of this paper. The contributions of this paper can be summarized as follows.

(1) In order to enhance the operability of the channel aware flipping method [10] in an energy constrained WSN, a specific energy limit indicator represented by the sensors’ activity probability is taken as the additional design constraint over the perfect secrecy. We call this modified scheme the transmission channel based only (TCBO) secure detection under energy constraint. Then, the simplified log-likelihood ratios (LLR) computed approximately under the low and high signal-to-noise ratio (SNR) conditions are derived. Following that, we obtain asymptotic error probabilities of the ally fusion center (AFC) at the worst and best noise situations with help of the central limit theorem (CLT). Next, the optimization problems with the perfect secrecy and energy constraint are established to find three comparison thresholds used in the randomly flipping operation. After simplifying the optimization target functions, the optimal thresholds are discussed and achieved. The above framework for error probability analysis and parameters optimization will also be taken as the mathematic approach in our newly designed scheme to solve for the main parameters.

(2) Considering local detection performance also affects the decision fusion evidently, we combine the local observation quality with the transmission channel information to design a more efficient hybrid scheme. Here, the energy constraint is satisfied by censoring the sensor with a less informative local LLR and transmission security is guaranteed through randomly flipping the local decisions based on the estimated channel gains. This innovative scheme is called the joint local decision and wireless transmission (JLDWT) scheme. Then, following the mathematic framework given by the first work, two local detection thresholds and one flipping comparison threshold are optimized to minimize the AFC’s error rates, besides, satisfy the perfect secrecy condition and the energy limitation.

(3) At last, through an overall simulation from diffident perspectives, the above two schemes are evaluated in a practical wireless transmission environment. The simulation results demonstrate that the new proposed hybrid scheme can improve the error performance of the AFC under a relatively high SNR transmission environment with a more severe energy constraint, as well as, maintain the perfect secrecy.

The rest of the paper is organized as follows: an overview of related work is discussed in Section 2. Section 3 describes the system model. The TCBO and JLDWT schemes are presented in Section 4 and Section 5, respectively. The simulation results are discussed in Section 6. Section 7 concludes the paper.

## 2. Related Work

In this section, we summarize the related work about physical layer security suitable for the IoT. The communication network consisting of controllers and actuators and the sensor network composed of sensors and controllers are two main subsystems of an abstracted IoT network [2]. The physical layer security solutions possibly available for both subsystems will be presented in the following text.

In the communication network of the IoT, the controllers are the signal transmitters, which could be equipped with multiple antennas and an adequate energy supply. Then, some of the classical secure schemes at physical layer proposed for the downlink in LTE-Advanced network may be usable [30,31,32,33,34,35,36,37,38,39]. When the main channel (the transmitter to legitimate receiver channel) and the eavesdropper channel are perfectly known, the beamforming (precoding) techniques can be adopted to maximize the signal quality difference between the destination and the eavesdropper by strengthening or weakening signals in certain dimensions. For the scenario of multiple-input, single-output and multi-antenna eavesdropper (MISOME) with a single legitimate receiver, the optimal beamforming vector is the generalized eigenvector corresponding to the largest generalized eigenvalue of the receiver and the eavesdropper channel covariance matrice [30]. While, under the multiple-input, multiple-output and multi-antenna eavesdropper (MIMOME) scenario, the search for the optimal precoder with a total power constraint has a non-convex form and the solution can be found numerically. If the power covariance constraint is considered, a closed form solution based on the generalized eigenvalue decomposition (GEVD) can be obtained [31]. As for the case of multiple receivers and eavesdroppers, the achievable secrecy rates can be used to build optimization problems to find a secrecy beamformer or precoder [32], further, a simpler but less effective design can be achieved using the channel inversion technique [33]. In addition, when the eavesdropper’s CSI is unknown, emitting artificial noise (AN) is helpful to prevent the eavesdropper from getting a good channel. The AN is often added in the null space of the main channel with single destination and eavesdropper [34]. While, for the case with multiple receivers and eavesdroppers, the AN would be placed in the null space of the effective channels of all receivers [35]. Since AN may reduce the transmission power of the useful data, power allocation between data and AN should be examined to ensure good performance under secrecy constraint [36]. Another novel strategy to degrade the eavesdropper’s channel quality is based on noise aggregation [40,41], where two adjacent timeslots are bounded to transmit two packets and the transmitter performs bitwise exclusive-or (XOR) operation on the even packet with previous odd one. Because the legitimate receiver can detect the packets in odd slots correctly by an ARQ protocol while eavesdropper may only have a noisy observation, the channel noise in odd slots is aggregated to even slots [41]. Obviously, many of the above security schemes are difficult to be directly employed in an IoT setting, because the accurate legitimate channel state information at the transmitter (CSIT) is difficult to acquire for the channel training opportunities are limited and the high rate feedback channels are lack in the IoT. Moreover, the eavesdropper CSIT is more difficult to yield since eavesdroppers remain completely passive. As for the AN based methods are also not desirable due to their higher energy expenditure [2].

In addition, a variety of physical layer security solutions have been proposed in literature for the distributed detection in sensor networks. With the assumption that the eavesdropping fusion center (EFC) can only distinguish busy-idle state of sensor’s transmission, an optimal sensor censoring scheme with a perfect secrecy and energy constraint was given in [8]. But the processing capability of the EFC was too limited. Another category of effective scheme is the probabilistic ciphering based one, where the sensor’s observation is randomly mapped to a set of quantization levels according to an optimal mapping probabilities matrix [9,24,25]. However, the security is assured by assuming the EFC being completely ignorant about the mapping probabilities. Moreover, the crucial energy efficient issue was not discussed. In [26,27], the optimal local quantizer was examined through minimizing the detection cost at the AFC meanwhile satisfying the constraints to the EFC detection cost or error performance, but the energy consumption problem was not concerned, either. In addition, all of the above solutions were not evaluated over a practical wireless channel and the effect of the transmission channel on their security was not discussed. Afterwards, a category of channel aware encryption method was proposed to realize the perfect secrecy from the EFC, including the type-based multiple access scheme proposed in [23] and the channel-based bit flipping scheme designed in [10], where not the accurate channel coefficients were needed, but only the channel gains had to be estimated using the pilot signal from the AFC. In channel aware encryption, the good energy efficiency could be realized through introducing the dormant sensors. The inherent significant difference of the wireless channels for the EFC and the AFC was explored to achieve the perfect secrecy of the sensor’s information transmission, due to the channels from sensors to the EFC and the AFC are independent of each other. Especially, the channel-based randomly flipping method is very suitable for the distributed detection due to its low complexity, good scalability and less limitation on the EFC. However, the work in [10] did not give an efficient solution to optimize three comparison thresholds. In addition, when the sleeping sensor was chosen, the channel gain was taken as the only metric while the local decision quality was not concerned although it may induce more important influence on the fusion performance. In addition, AN based mechanisms that let a part of sensors or the AFC transmit the jamming signal to degrade the SINR of the EFC were also introduced to the sensor network [28,29]. However, the performance of the AFC would also be reduced when the jamming signal worsens the EFC channel [29] or the external energy would be spent by the AFC to interfere the EFC [28]. Based on the above drawbacks of the previous works, we propose the secure and energy efficient JLDWT scheme, which is a hybrid method combing the local detection and the wireless transmission, after designing an analysis framework to complete the performance analysis and thresholds optimization of TCBO scheme.

## 3. System Model

In this section, the concerned IoT sensor network scenario is given. The local detection and the transmission scheme of local decisions from the sensors to the fusion center are introduced.

### 3.1. IoT Sensor Network

**Figure 1:**
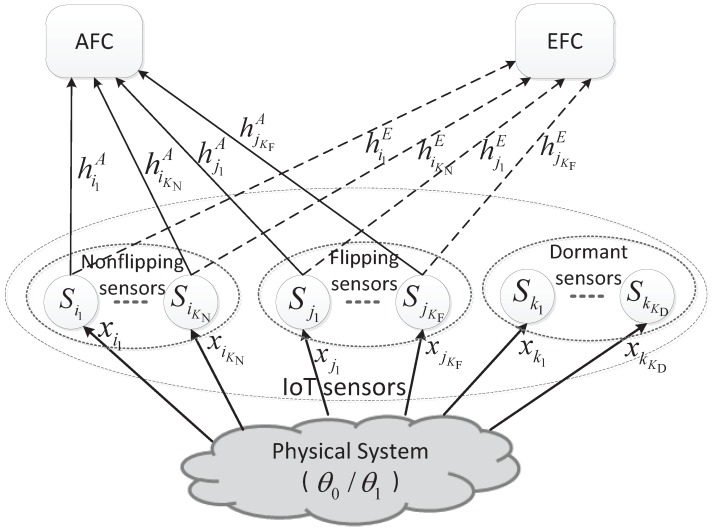
IoT sensor network with the ally fusion center and eavesdropping fusion center.

Consider a sensor network in IoT system illustrated by Figure 1, which performs distributed detection for a binary hypothesis test of $\theta_{0}$ against $\theta_{1}$. A number of sensors are distributed near the physical system to detect a binary target state and transmit their local decision results to an AFC through a wireless parallel access channel (PAC). Meanwhile, a passive EFC overhears the communications between the sensors and AFC and also attempts to detect the state of *θ*. The channels from sensors to the AFC and the EFC are called the main and eavesdropping channels, respectively. Moreover, the concerned sensor network is energy-constrained for the power supplies of the sensors are usually severely constrained. Obviously, the security and energy saving are the main challenges faced by our senor network. Therefore, in each local decision reporting slot, some sensors will keep dormant to meet the energy constraint and some sensors among the active ones will transmit the bit-flipping version of local detection results to make the EFC confused.

In Figure 1, the sensors with the indices in the sets of $\left\{i_{1},i_{2},\ldots ,i_{K_{N}}\right\}$, $\left\{j_{1},j_{2},\ldots ,j_{K_{F}}\right\}$ and $\left\{k_{1},k_{2},\ldots ,k_{K_{D}}\right\}$ are included in the non-flipping group, flipping group and the dormant group, respectively. Thus, the total number of sensors in the network is $K=K_{F}+K_{D}+K_{N}$. In addition, the observation to the physical system of the *k*-th sensor is denoted by $x_{k}$. The communication channels from sensors to the AFC and the EFC are represented by $h_{k}^{A}$ and $h_{k}^{E}$, respectively. And they are assumed to be independent and identically distributed (i.i.d.) Rayleigh block fading channels. Moreover, a transmission probability or an activation probability *β*, which is proportional to the per-sensor energy consumption, is introduced to represent the energy constraint.

### 3.2. Local Detection of Sensors

For the *k*-th sensor, the acquired observation corrupted by additive noise is modeled as:

(1) $$\begin{array}{c} \theta_{0}:\;\;\;\;\;x_{k}=w_{k}\\ \theta_{1}:\;\;\;\;\;x_{k}=\theta +w_{k} \end{array}$$

where $w_{k}$ is an i.i.d zero-mean Gaussian random variable with variance $\sigma^{2}$, i.e., $w_{k}∼N\left(0,\sigma^{2}\right)$. Thus the SNR of local detection can be computed and denoted by $snr_{L}=\theta^{2}/\sigma^{2}$. Based on the observation, the sensor makes a one-bit local decision $b_{k}\in \left\{0,1\right\}$ to indicate the absence or presence of *θ* by using the Bayesian detection criteria:

(2) $$\frac{f(\left.\theta_{1}\right|x_{k})}{f(\left.\theta_{0}\right|x_{k})}\begin{array}{c} \overset{b_{k}=1}{>}\lambda_{U}\\ \underset{b_{k}=0}{<}\lambda_{L} \end{array}$$

where $f(\left.\theta_{i}\right|x_{k})$ is the posterior probability distribution function (PDF) of $\theta_{i}$ based on $x_{k}$ for i=0,1. The main difference of Equation (2) from the traditional Bayesian detection is that two rather than one local decision thresholds are set here. $\lambda_{U}$ and $\lambda_{L}$, which meet $0<\lambda_{L}\leq \lambda_{U}<\infty$, are the upper and lower thresholds and assumed to be identical at all the sensors. If the ratio of the posterior probability distribution lies inside the region of $\left[\lambda_{L},\lambda_{U}\right]$, it means that the observation appears less informative for discriminating between $\theta_{0}$ and $\theta_{1}$, so the corresponding decision result is more likely to be false. As for such kind of sensors, it is better to keep them silent for energy efficiency. Of course, this is the basic idea of the sensor censoring technique [8,42]. However, in this paper, we adopt it to realize the energy saving for the secure transmission of sensors and the details are described in Section 5.

The prior probabilities of $\theta_{0}$ and $\theta_{1}$ are assumed to be $q_{0}$ and $q_{1}$, respectively. Then the Equation (2) can be transformed into:

(3) $$\lambda_{k}=\frac{f(\left.x_{k}\right|\theta_{1})}{f(\left.x_{k}\right|\theta_{0})}\begin{array}{c} \overset{b_{k}=1}{>}\lambda_{U}\left(q_{0}/q_{1}\right)\\ \underset{b_{k}=0}{<}\lambda_{L}\left(q_{0}/q_{1}\right) \end{array}$$

where $f(\left.x_{k}\right|\theta_{i})$ is the conditional PDF of $x_{k}$ under the hypothesis $\theta_{i}$, and $\lambda_{k}$ is the likelihood ratio (LR). From Equation (1), it can be obtained that

(4) $$\begin{array}{c} f\left(\left.x_{k}\right|\theta_{1}\right)=\frac{\exp[-{\left(x_{k}-\theta \right)}^{2}/2\sigma^{2}]}{\sqrt{2\pi }\sigma }\\ f\left(\left.x_{k}\right|\theta_{0}\right)=\frac{\exp[-{\left(x_{k}\right)}^{2}/2\sigma^{2}]}{\sqrt{2\pi }\sigma } \end{array}$$

Furthermore, the log-likelihood ratio (LLR) can be written as

(5) $$\Lambda_{k}^{L}=\log\left(\lambda_{k}\right)=\frac{\theta }{\sigma^{2}}x_{k}-\frac{\theta^{2}}{2\sigma^{2}}$$

Combining Equations (4) and (5), it can be easily derived that the conditional PDFs of $\Lambda_{k}^{L}$ are

(6) $$\begin{array}{c} f\left(\left.\Lambda_{k}^{L}\right|\theta_{1}\right)=\frac{1}{\sqrt{2\pi \cdot snr_{L}}}\exp\left(-\frac{{\left(\Lambda_{k}^{L}-snr_{L}/2\right)}^{2}}{2\cdot snr_{L}}\right)\\ f\left(\left.\Lambda_{k}^{L}\right|\theta_{0}\right)=\frac{1}{\sqrt{2\pi \cdot snr_{L}}}\exp\left(-\frac{{\left(\Lambda_{k}^{L}+snr_{L}/2\right)}^{2}}{2\cdot snr_{L}}\right) \end{array}$$

Furthermore, we can obtain that the equation $f\left(\left.\Lambda_{k}^{L}\right|\theta_{1}\right)/f\left(\left.\Lambda_{k}^{L}\right|\theta_{0}\right)=\exp\left(\Lambda_{k}^{L}\right)$ is satisfied and this is the nesting property of the LR.

There are four possible cases for local detection, namely correct decisions under two states, missed detection and false alarm. Based on Equations (3) and (6), we can calculate the probabilities of four cases and obtain

(7) $$\begin{array}{c} P_{d}=\int_{\log(\lambda_{U}\cdot q_{0}/q_{1})}^{\infty }f\left(\Lambda_{k}^{L}|\theta_{1}\right)d\Lambda_{k}^{L}=Q\left(\frac{log\left(\lambda_{U}\cdot q_{0}/q_{1}\right)-snr_{L}/2}{\sqrt{snr_{L}}}\right)\\ P_{m}=\int_{-\infty }^{\log(\lambda_{L}\cdot q_{0}/q_{1})}f\left(\Lambda_{k}^{L}|\theta_{1}\right)d\Lambda_{k}^{L}=1-Q\left(\frac{\log\left(\lambda_{L}\cdot q_{0}/q_{1}\right)-snr_{L}/2}{\sqrt{snr_{L}}}\right)\\ P_{f}=\int_{\log(\lambda_{U}\cdot q_{0}/q_{1})}^{+\infty }f\left(\Lambda_{k}^{L}|\theta_{0}\right)d\Lambda_{k}^{L}=Q\left(\frac{\log\left(\lambda_{U}\cdot q_{0}/q_{1}\right)+snr_{L}/2}{\sqrt{snr_{L}}}\right)\\ P_{0d}=\int_{-\infty }^{\log(\lambda_{L}\cdot q_{0}/q_{1})}f\left(\Lambda_{k}^{L}|\theta_{0}\right)d\Lambda_{k}^{L}=1-Q\left(\frac{\log\left(\lambda_{L}\cdot q_{0}/q_{1}\right)+snr_{L}/2}{\sqrt{snr_{L}}}\right) \end{array}$$

where $P_{0d}$ is the probability of correct detection under *θ* being non-existent and $Q\left(x\right)\;=\;1/\sqrt{2\pi }\int_{x}^{\infty }\exp(-t^{2}/2)dt$. In addition, the error probability of local detection for each sensor can be defined as $P_{E_{L}}=q_{0}P_{f}+q_{1}P_{m}$. If we set $\lambda_{U}=\lambda_{L}=\lambda$, this error probability can be given by

(8) $$P_{E_{L}}=q_{0}Q\left(\frac{\log\left(\lambda \cdot q_{0}/q_{1}\right)+snr_{L}/2}{\sqrt{snr_{L}}}\right)+q_{1}\left[1-Q\left(\frac{\log\left(\lambda \cdot q_{0}/q_{1}\right)-snr_{L}/2}{\sqrt{snr_{L}}}\right)\right]$$

Furthermore, the first-order derivation of $P_{E_{L}}$ with respect to *λ* is

(9) $$\begin{array}{c} \frac{dP_{E_{L}}}{d\lambda }=\frac{l}{\lambda \cdot \sqrt{2snr_{L}}}\exp\left(-\frac{{\left[\log\left(\lambda \cdot q_{0}/q_{1}\right)\right]}^{2}+{\left(snr_{L}\right)}^{2}/4}{2snr_{L}}\right)\cdot \left(\frac{q_{1}}{\sqrt{\pi }}\exp\left[\frac{\log(\lambda \cdot q_{0}/q_{1})}{2}\right]-\frac{q_{0}}{\sqrt{\pi }}\exp\left[-\frac{\log(\lambda \cdot q_{0}/q_{1})}{2}\right]\right) \end{array}$$

Through letting $\frac{dP_{E_{L}}}{d\lambda }=0$, it can be obtained that the optimized $\lambda^{*}$ meeting 0<λ<∞ to minimize $P_{E_{L}}$ is $\lambda^{*}=1$.

### 3.3. Transmission of Local Decisions from Sensors to FC

After the local decisions are achieved, the sensors would deliver them to the AFC. In this paper, a wireless PAC between the sensors and the AFC is considered and the transmission channels from different sensors to the fusion center are orthogonal. However, the sensors’ transmissions are overheard by the EFC, who also wishes to detect the target state. From the literature [2,7,9], we have seen that the stochastic ciphering could be employed to protect the information of the sensors from the EFC efficiently, since each sensor would flip its decision randomly and the EFC would be confused when it was ignorant about the flipping probability (i.e., the encryption key). However, the key exchange between the AFC and the sensor itself may be not secure from the EFC. In this case, the channel-aware stochastic cipher [10], whose seeds are based on the randomness of the transmission channels, are preferable. Because the channels to the AFC and the EFC from a sensor are independent, it is impossible for the EFC to deterministically know the flipping action of a sensor based on the main channel gain. Thus, the formation leaked to the EFC reduces, although the flipping probability is completely known by the EFC. Therefore, the channel-based stochastic ciphering is still adopted by us to realize the secure transmission of local decisions from sensors to the AFC.

In order to sense the channel information, the sensors would firstly receive the known pilot signal from the AFC, as well as three thresholds for comparison. Then the estimated channel gain would be compared with the thresholds to determine which action should be selected by a sensor. The sensor may report an unaltered local decision, a “flipped” decision, or stay dormant to satisfy the energy constraint.

**Figure 2:**
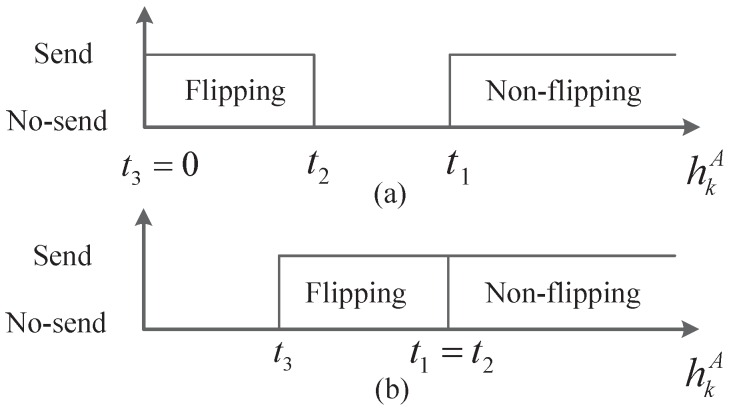
Single “No-send” region: (**a**) Case of $t_{3}=0$; (**b**) Case of $t_{1}=t_{2}$.

Assume the main channel and the eavesdropping channel both follow the Rayleigh distribution with unit power, i.e., $f\left(h\right)=2h\exp\left(-h^{2}\right)$ and h∈[0,∞), which is usually considered in existing studies [10,23,42]. Assume the pilot signal is so strong that the sensors can obtain the exact channel gains. Basing on the channel reciprocity, the sensors’ estimated channel gains can be used to indicate the sensor-to-AFC channels. Moreover, they are unknown by the EFC due to the statistical independence of the main channel and the eavesdropping channel. The thresholds broadcasted by the AFC are $\left\{t_{1},t_{2},t_{3}\right\}$ with $0\leq t_{3}\leq t_{2}\leq t_{1}<\infty$. Thus, the secure transmission strategy with energy limitation is that, sensor *k* reports its original local decision if $h_{k}^{A}>t_{1}$, reports a bit-flipping decision if $t_{3}\leq h_{k}^{A}\leq t_{2}$ and stays silent for energy efficiency otherwise. From the security analysis given in [10], we can see that the condition for perfect secrecy is $\lambda_{1}\overset{\mathrm{def}}{=}\int_{t_{1}}^{\infty }f\left(h_{k}^{A}\right)dh_{k}^{A}=\int_{t_{3}}^{t_{2}}f\left(h_{k}^{A}\right)dh_{k}^{A}\overset{\mathrm{def}}{=}\lambda_{2}$. Obviously, to meet the energy constraint of network, the inequality of $\lambda_{1}+\lambda_{2}\leq \beta$ should also be held. Moreover, the case with a single “no-send” region is concerned in this paper. That is to say either $t_{3}=0$ or $t_{1}=t_{2}$, which is illustrated in Figure 2.

## 4. Transmission Channel Based Only Secure Detection under Energy Constraint

In [10], the authors designed a confidential and energy efficient distributed detection method, called channel aware encryption, only from the view of the wireless transmission between sensors and the fusion center. And the condition for perfect secrecy was derived. Moreover, the LLR based decision fusion was studied, further, a simplified decision fusion rule in high SNR region was given. However, the more detailed analysis about the error probability of decision fusion and the optimization of thresholds were absent. In this section, we will analyze the error performance of the AFC based on the approximated LLRs derived under low and high SNR conditions, respectively. Afterwards, three thresholds will be optimized to minimize the probability of error at the AFC while ensuring the perfect secrecy from the EFC and satisfying the energy constraint. It should be noted that a specified energy constraint of β≤1 is introduced by us. And the adjusted scheme is called the TCBO secure detection under energy constraint in our paper.

### 4.1. Approximation of LLR and Error Probabilities of FC

For the secure scheme only basing on transmission channels, the confidentiality from the eavesdropper and the energy saving are both provided by the reporting strategy of local decisions. Thus, the thresholds used in the local detection are set as $\lambda_{L}=\lambda_{U}=\lambda^{*}$ to optimize the sensor’s local performance. Then, we have $P_{m}=1-P_{d}$ and $P_{0d}=1-P_{f}$. In addition, the common binary phase shift keying (BPSK) modulation is utilized by each sensor to deliver its one-bit decision. At the fusion center, the LLR based fusion rule is used and the transmission channel information is unknown. In addition, it is assumed that the fusion rules and the Prior information at the EFC are identical with those at the AFC and this is a worst case from the view of security.

The received signals at the AFC and EFC from sensor *k* are denoted as $y_{k}^{A}$ and $y_{k}^{E}$, respectively. They can be described as

(10) $$\begin{array}{c} y_{k}^{A}=h_{k}^{A}x_{k}+n_{k}^{A}\\ y_{k}^{E}=h_{k}^{E}x_{k}+n_{k}^{E} \end{array}$$

where $n_{k}^{A}∼N\left(0,\delta_{A}^{2}\right)$ and $n_{k}^{E}∼N\left(0,\delta_{E}^{2}\right)$. Thus, the transmission channel SNR for the AFC and EFC can be written as $SNR_{A}={\left|h_{k}^{A}x_{k}\right|}^{2}/\delta_{A}^{2}$ and $SNR_{E}={\left|h_{k}^{E}x_{k}\right|}^{2}/\delta_{E}^{2}$, respectively. Following the channel-aware flipping rule, we have $x_{k}=2b_{k}-1$ for $h_{k}^{A}>t_{1}$, $x_{k}=2{\bar{b}}_{k}-1$ for $t_{3}\leq h_{k}^{A}\leq t_{2}$ and $x_{k}=0$ for other $h_{k}^{A}$. The LLR at the AFC can be expressed in terms of $y^{A}=\left[y_{1}^{A},y_{2}^{A},...,y_{K}^{A}\right]$ as

(11) $$\Lambda^{A}=\frac{1}{K}\log\frac{f\left(y^{A}|\theta_{1}\right)}{f\left(y^{A}|\theta_{0}\right)}\overset{\left(a\right)}{=}\frac{1}{K}\sum_{k=1}^{K}\log\frac{f\left(y_{k}^{A}|\theta_{1}\right)}{f\left(y_{k}^{A}|\theta_{0}\right)}$$

where (a) is due to the independence of different $y_{k}^{A}$ and $f\left(y_{k}^{A}|\theta_{i}\right)$ denotes the likelihood function of sensor *k* for the hypothesis $\theta_{i}$. For the Bayesian setup, the optimal decision rule can be given by $\Lambda^{A}\begin{array}{c} \overset{\theta_{1}}{<}\\ \underset{\theta_{0}}{>} \end{array}$ log(*q*0/*q*1).

By using the similar derivation method in Section IV of [10], it can be achieved

(12) $$\begin{array}{cc} f\left(y_{k}^{A}|\theta_{i}\right) & =P\left(b_{k}=1|\theta_{i}\right)\left[\Phi \left(t_{1},\infty ,1,y_{k}^{A},\delta_{A}^{2}\right)+\Phi \left(t_{3},t_{2},-1,y_{k}^{A},\delta_{A}^{2}\right)\right]\\ & +P\left(b_{k}=0|\theta_{i}\right)\left[\Phi \left(t_{1},\infty ,-1,y_{k}^{A},\delta_{A}^{2}\right)+\Phi \left(t_{3},t_{2},1,y_{k}^{A},\delta_{A}^{2}\right)\right]\\ & +\left[\Phi \left(0,t_{3},0,y_{k}^{A},\delta_{A}^{2}\right)+\Phi \left(t_{2},t_{1},0,y_{k}^{A},\delta_{A}^{2}\right)\right] \end{array}$$

where

(13) $$\begin{array}{cc} \Phi \left(t_{a},t_{b},x_{k},y_{k}^{A},\delta_{A}^{2}\right) & =\int_{t_{a}}^{t_{b}}f\left(y_{k}^{A}|h_{k}^{A},x_{k}\right)f\left(h_{k}^{A}\right)dh_{k}^{A}\\ & =\int_{t_{a}}^{t_{b}}\frac{1}{\sqrt{2\pi }\delta_{A}}\exp\left(-\frac{{\left(y_{k}^{A}-h_{k}^{A}x_{k}\right)}^{2}}{2\delta_{A}^{2}}\right)2h_{k}^{A}\exp\left(-h{{}_{k}^{A}}^{2}\right)dh_{k}^{A} \end{array}$$

Note that the LLR based on Equation (12) requires numerical integrations. It is greatly unfavorable to the performance analysis of decision fusion and the optimization of comparison thresholds. Therefore, the approximations of LLR under low SNR and high SNR scenarios would be examined. Moreover, the error probabilities based on these approximations would be analyzed in follows.

#### 4.1.1. Approximation of LLR and Error Performance under Low SNR

As the channel noise variance $\delta_{A}^{2}\to \infty$, we can get

(14) $$\begin{array}{cc} \Phi \left(t_{a},t_{b},x_{k},y_{k}^{A},\delta_{A}^{2}\right) & \approx N\left(y_{k}^{A},\delta_{A}^{2}\right)\{exp\left(-t_{a}^{2}\right)-exp\left(-t_{b}^{2}\right)\\ & +\frac{y_{k}^{A}x_{k}}{\delta_{A}^{2}}\left[t_{a}exp\left(-t_{a}^{2}\right)-t_{b}exp\left(-t_{b}^{2}\right)+\int_{t_{a}}^{t_{b}}exp(-h^{2})dh\right]\} \end{array}$$

where $N\left(y_{k}^{A},\delta_{A}^{2}\right)=1/\left(\sqrt{2\pi }\delta_{A}\right)\exp\left[-{\left(y_{k}^{A}\right)}^{2}/\left(2\delta_{A}^{2}\right)\right]$. The detailed derivation of Equation (14) is given in the Appendix A. Applying Equation (14) to Equation (12), it can be obtained that

(15) $$\begin{array}{cc} f\left(y_{k}^{A}|\theta_{1}\right) & =N\left(y_{k}^{A},\delta_{A}^{2}\right)\cdot \\ & \{\left[\Phi \left(t_{1},\infty ,-1,y_{k}^{A},\delta_{A}^{2}\right)+\Phi \left(t_{3},t_{2},1,y_{k}^{A},\delta_{A}^{2}\right)\;+\Phi \left(0,t_{3},0,y_{k}^{A},\delta_{A}^{2}\right)+\Phi \left(t_{2},t_{1},0,y_{k}^{A},\delta_{A}^{2}\right)\right]\\ & +P_{d}\left[\Phi \left(t_{1},\infty ,1,y_{k}^{A},\delta_{A}^{2}\right)-\Phi \left(t_{1},\infty ,-1,y_{k}^{A},\delta_{A}^{2}\right)\;+\Phi \left(t_{3},t_{2},-1,y_{k}^{A},\delta_{A}^{2}\right)-\Phi \left(t_{3},t_{2},1,y_{k}^{A},\delta_{A}^{2}\right)\right]\}\\ & \approx N\left(y_{k}^{A},\delta_{A}^{2}\right)\left\{1+\frac{y_{k}^{A}}{\delta_{A}^{2}}\left[m\left(t_{1}\right)-n\left(t_{3},t_{2}\right)\right]\left(2P_{d}-1\right)\right\} \end{array}$$

(16) $$f\left(y_{k}^{A}|\theta_{0}\right)=N\left(y_{k}^{A},\delta_{A}^{2}\right)\left\{1+\frac{y_{k}^{A}}{\delta_{A}^{2}}\left[m\left(t_{1}\right)-n\left(t_{3},t_{2}\right)\right]\left(2P_{f}-1\right)\right\}$$

where

(17) $$\begin{array}{c} m\left(t_{1}\right)=t_{1}\exp\left(-t_{1}^{2}\right)+\int_{t_{1}}^{\infty }exp(-h^{2})dh\\ n\left(t_{3},t_{2}\right)=t_{3}\exp\left(-t_{3}^{2}\right)-t_{2}\exp\left(-t_{2}^{2}\right)+\int_{t_{3}}^{t_{2}}exp(-h^{2})dh \end{array}$$

From Equations (15) and (16), we achieve

(18) $$\begin{array}{cc} \Lambda_{k}^{A} & =\log\frac{f\left(y_{k}^{A}|\theta_{1}\right)}{f\left(y_{k}^{A}|\theta_{0}\right)}=\log\left[1+\frac{f\left(y_{k}^{A}|\theta_{1}\right)-f\left(y_{k}^{A}|\theta_{0}\right)}{f\left(y_{k}^{A}|\theta_{0}\right)}\right]\\ & \approx \log\{1+\frac{2\left(P_{d}-P_{f}\right)\frac{y_{k}^{A}}{\delta_{A}^{2}}\left[m\left(t_{1}\right)-n\left(t_{3},t_{2}\right)\right]}{1+\left(2P_{f}-1\right)\frac{y_{k}^{A}}{\delta_{A}^{2}}\left[m\left(t_{1}\right)-n\left(t_{3},t_{2}\right)\right]}\} \end{array}$$

Following the assumption of $\delta_{A}^{2}\to \infty$ and the fact that log(1+x)≈x with *x* closing to zero, we can further reduce Equation (18) to

(19) $$\begin{array}{cc} \Lambda_{k}^{A} & \approx 2\left(P_{d}-P_{f}\right)\frac{\left[m\left(t_{1}\right)-n\left(t_{3},t_{2}\right)\right]}{\delta_{A}^{2}}y_{k}^{A}\\ & =\Gamma \left(\lambda^{*},t_{3},t_{2},t_{1}\right)\cdot y_{k}^{A} \end{array}$$

From Equation (19), we can see that the calculation of LLR can be simplified significantly for large noise variance. Note that the formulas from Equation (11) to Equation (19) are also available for the EFC provided it has the same prior information as the AFC. The only variation is the different received signal $y_{k}^{E}$ from $y_{k}^{A}$.

Since $y_{k}^{A}$ is independent from each other, $\Lambda^{A}=\frac{1}{K}\sum_{k=1}^{K}\Lambda_{k}^{A}$ can be taken as the average of *K* i.i.d. random variables. Then, invoking the central limit theorem [9,23], we can deem that the statistic of $\Lambda^{A}$ converges to a normal distribution for a large *K*. That is $\Lambda^{A}|\theta_{i}∼N\left(\mu_{Ak}|\theta_{i},\frac{\gamma_{Ak}^{2}|\theta_{i}}{K}\right)$, where $\mu_{Ak}|\theta_{i}$ and $\gamma_{Ak}^{2}|\theta_{i}$ are the mean and variance of $\Lambda_{k}^{A}$ conditioned on $\theta_{i}$, respectively. And they are directly related with the mean and the variance of $y_{k}^{A}$, which can be seen from Equation (19). Next, our target is to calculate $E\left(y_{k}^{A}|\theta_{i}\right)$ and $Var\left(y_{k}^{A}|\theta_{i}\right)$.

Utilizing Equation (15), we can write

(20) $$\begin{array}{c} E\left(y_{k}^{A}|\theta_{1}\right)=\int_{-\infty }^{+\infty }y_{k}^{A}f\left(y_{k}^{A}|\theta_{1}\right)dy_{k}^{A}\\ \;\;\;\;\;\;\;\;\;\;\;\;\;\;\;\;\approx \int_{-\infty }^{+\infty }y_{k}^{A}N\left(y_{k}^{A},\delta_{A}^{2}\right)dy_{k}^{A}+\frac{\left[m\left(t_{1}\right)-n\left(t_{3},t_{2}\right)\right]\left(2P_{d}-1\right)}{\delta_{A}^{2}}\int_{-\infty }^{+\infty }{\left(y_{k}^{A}\right)}^{2}N\left(y_{k}^{A},\delta_{A}^{2}\right)dy_{k}^{A}\\ \;\;\;\;\;\;\;\;\;\;\;\;\;\;\;\;\overset{\left(a\right)}{=}\left[m\left(t_{1}\right)-n\left(t_{3},t_{2}\right)\right]\left(2P_{d}-1\right) \end{array}$$

(21) $$E\left(y_{k}^{A}|\theta_{0}\right)=\left[m\left(t_{1}\right)-n\left(t_{3},t_{2}\right)\right]\left(2P_{f}-1\right)$$

where (a) is due to $\int_{-\infty }^{+\infty }y_{k}^{A}N\left(y_{k}^{A},\delta_{A}^{2}\right)dy_{k}^{A}=0$ and $\int_{-\infty }^{+\infty }{\left(y_{k}^{A}\right)}^{2}N\left(y_{k}^{A},\delta_{A}^{2}\right)dy_{k}^{A}=\delta_{A}^{2}$, whose derivations are described in Appendix B.

In order to obtain $Var\left(y_{k}^{A}|\theta_{i}\right)$, we firstly calculate

(22) $$\begin{array}{c} E\left({\left(y_{k}^{A}\right)}^{2}|\theta_{1}\right)=\int_{-\infty }^{+\infty }{\left(y_{k}^{A}\right)}^{2}f\left(y_{k}^{A}|\theta_{1}\right)dy_{k}^{A}\\ \;\;\;\;\;\;\;\;\;\;\;\;\;\;\;\;\approx \int_{-\infty }^{+\infty }{\left(y_{k}^{A}\right)}^{2}N\left(y_{k}^{A},\delta_{A}^{2}\right)dy_{k}^{A}+\frac{\left(2P_{d}-1\right)}{\delta_{A}^{2}}\left[m\left(t_{1}\right)-n\left(t_{3},t_{2}\right)\right]\int_{-\infty }^{+\infty }{\left(y_{k}^{A}\right)}^{3}N\left(y_{k}^{A},\delta_{A}^{2}\right)dy_{k}^{A}\\ \;\;\;\;\;\;\;\;\;\;\;\;\;\;\;\overset{\left(a\right)}{=}\delta_{A}^{2} \end{array}$$

where (a) follows the fact of $\int_{-\infty }^{+\infty }{\left(y_{k}^{A}\right)}^{3}N\left(y_{k}^{A},\delta_{A}^{2}\right)dy_{k}^{A}=0$ verified also in Appendix B. Obviously, $E\left({\left(y_{k}^{A}\right)}^{2}|\theta_{0}\right)=\delta_{A}^{2}$. Then $Var\left(y_{k}^{A}|\theta_{i}\right)$ can be achieved through $Var\left(y_{k}^{A}|\theta_{i}\right)=E\left({\left(y_{k}^{A}\right)}^{2}|\theta_{i}\right)-E^{2}\left(y_{k}^{A}|\theta_{i}\right)$.

Combing Equations (19)∼(22), along with the Bayesian decision rule, we can yield the error probability for the AFC as follows:

(23) $$\begin{array}{cc} P_{e}^{A} & =q_{0}P\left(\Lambda^{A}\geq \log\left(q_{0}/q_{1}\right)\left|\theta_{0}\right.\right)+q_{1}P\left(\Lambda^{A}<\log\left(q_{0}/q_{1}\right)\left|\theta_{1}\right.\right)\\ & =q_{0}Q\left(\frac{\log\left(q_{0}/q_{1}\right)-\Gamma \left(\lambda^{*},t_{3},t_{2},t_{1}\right)E\left(y_{k}^{A}|\theta_{0}\right)}{\sqrt{\Gamma^{2}\left(\lambda^{*},t_{3},t_{2},t_{1}\right)\left[\delta_{A}^{2}-E^{2}\left(y_{k}^{A}|\theta_{0}\right)\right]/K}}\right)\\ & +q_{1}\left[1-Q\left(\frac{\log\left(q_{0}/q_{1}\right)-\Gamma \left(\lambda^{*},t_{3},t_{2},t_{1}\right)E\left(y_{k}^{A}|\theta_{1}\right)}{\sqrt{\Gamma^{2}\left(\lambda^{*},t_{3},t_{2},t_{1}\right)\left[\delta_{A}^{2}-E^{2}\left(y_{k}^{A}|\theta_{1}\right)\right]/K}}\right)\right] \end{array}$$

Clearly, the error probability for large $\delta_{A}^{2}$ has been expressed as a function of some specific parameters, namely $\lambda^{*},t_{3},t_{2},t_{1}$ and $\delta_{A}^{2}$. In Section 4.2, this asymptotic error probability would be taken as the optimization objection for finding the optimal comparison thresholds.

#### 4.1.2. Approximation of LLR and Error Performance under High SNR

Considering the high SNR scenario, i.e., $\delta_{A}^{2}\to 0$, we derive a simplified LLR referring to the idea of [10]. Assume the FC can estimate the instantaneous sensor-to-FC channel gain as ${\hat{h}}_{k}^{A}=\left|y_{k}^{A}\right|$ since $y_{k}^{A}\approx h_{k}^{A}x_{k}$ and $\left|x_{k}\right|=1$ except under the dormant case. Then, a simple hard decision rule determining which one a received signal $y_{k}^{A}$ comes from among three groups can be realized. A hard decision threshold $t_{h}$ is selected to satisfy $\int_{\tau_{3}}^{t_{h}}f\left(h_{k}^{A}\right)dh_{k}^{A}=\int_{t_{h}}^{\infty }f\left(h_{k}^{A}\right)dh_{k}^{A}$. Thus, the following conditional probability can reduce to

(24) $$p\left(x_{k}|b_{k}\right)=\left\{\begin{array}{cc} \delta_{x_{k},\left(2b_{k}-1\right)} & {\hat{h}}_{k}^{A}\geq t_{h}\\ \delta_{-x_{k},\left(2b_{k}-1\right)} & {\hat{h}}_{k}^{A}<t_{h} \end{array}\right.$$

where $\delta_{x,b}$ is the Kronecker delta function. Thus, the likelihood function $f\left(y_{k}^{A}|\theta_{i}\right)$ can be calculated as

(25) $$\begin{array}{c} f\left(y_{k}^{A}|\theta_{i}\right)\\ \;\;\;\;\;\;\;\;=\sum_{b_{k}}p\left(b_{k}|\theta_{i}\right)\sum_{x_{k}}f\left(y_{k}^{A}|x_{k},{\hat{h}}_{k}^{A}\right)p\left(x_{k}|b_{k}\right)\\ \;\;\;\;\;\;\;\;=\left\{\begin{array}{c} p\left(b_{k}=1|\theta_{i}\right)f\left(y_{k}^{A}|x_{k}=1,{\hat{h}}_{k}^{A}\right)+p\left(b_{k}=0|\theta_{i}\right)f\left(y_{k}^{A}|x_{k}=-1,{\hat{h}}_{k}^{A}\right),\;\;\;\;\;\;{\hat{h}}_{k}^{A}\geq t_{h}\\ p\left(b_{k}=1|\theta_{i}\right)f\left(y_{k}^{A}|x_{k}=-1,{\hat{h}}_{k}^{A}\right)+p\left(b_{k}=0|\theta_{i}\right)f\left(y_{k}^{A}|x_{k}=1,{\hat{h}}_{k}^{A}\right),\;\;\;\;\;\;{\hat{h}}_{k}^{A}<t_{h} \end{array}\right. \end{array}$$

Further derivation whose detail is provided in Appendix C gives that

(26) $$\Lambda_{k}^{A}=\left\{\begin{array}{c} 0,\;\;\;\;\;\;\;\;\;\;\;\;\;\;\;\;\;\;\;\;\;\;\;\;\;\;\;\;\;\;\;\;\;\;\;\;y_{k}^{A}=0\\ \log\frac{P_{d}}{P_{f}},\;\;\;\;\;\;\;\;{\hat{h}}_{k}^{A}\geq t_{h}\cap y_{k}^{A}>0\\ \log\frac{1-P_{d}}{1-P_{f}},{\hat{h}}_{k}^{A}\geq t_{h}\cap y_{k}^{A}<0\\ \log\frac{1-P_{d}}{1-P_{f}},{\hat{h}}_{k}^{A}<t_{h}\cap y_{k}^{A}>0\\ \log\frac{P_{d}}{P_{f}},\;\;\;\;\;\;\;\;{\hat{h}}_{k}^{A}<t_{h}\cap y_{k}^{A}<0 \end{array}\right.$$

Replacing $y_{k}^{A}$ and $\;{\hat{h}}_{k}^{A}$ with $y_{k}^{E}$ and $\;{\hat{h}}_{k}^{E}$ in Equation (26), the simplified LLR under high SNR for the EFC is got.

In order to yield the error probability, the mean and variance of $\Lambda_{k}^{A}$ are needed when the CLT is still used. Because $h_{k}^{A}\geq 0$, we have $y_{k}^{A}>0$ is equivalent to $x_{k}=1$ and $y_{k}^{A}<0$ corresponds to $x_{k}=-1$. Further, with the assumption of $h_{k}^{A}\approx {\hat{h}}_{k}^{A}$, it can be derived

(27) $$\begin{array}{c} E\left(\Lambda_{k}^{A}|\theta_{1}\right)=\left(\lambda_{1}+\lambda_{2}\right)\left[P_{d}\log\frac{P_{d}}{P_{f}}+\left(1-P_{d}\right)\log\frac{1-P_{d}}{1-P_{f}}\right]\\ E\left(\Lambda_{k}^{A}|\theta_{0}\right)=\left(\lambda_{1}+\lambda_{2}\right)\left[P_{f}\log\frac{P_{d}}{P_{f}}+\left(1-P_{f}\right)\log\frac{1-P_{d}}{1-P_{f}}\right] \end{array}$$

(28) $$\begin{array}{c} E\left[{\left(\Lambda_{k}^{A}\right)}^{2}|\theta_{1}\right]=\left(\lambda_{1}+\lambda_{2}\right)\left[P_{d}{\left(\log\frac{P_{d}}{P_{f}}\right)}^{2}+\left(1-P_{d}\right){\left(\log\frac{1-P_{d}}{1-P_{f}}\right)}^{2}\right]\\ E\left[{\left(\Lambda_{k}^{A}\right)}^{2}|\theta_{0}\right]=\left(\lambda_{1}+\lambda_{2}\right)\left[P_{f}{\left(\log\frac{P_{d}}{P_{f}}\right)}^{2}+\left(1-P_{f}\right){\left(\log\frac{1-P_{d}}{1-P_{f}}\right)}^{2}\right] \end{array}$$

The derivations of Equations (27) and (28) are referred to Appendix D. Moreover, applying Equations (27) and (28) to calculate the error probability obtains

(29) $$\begin{array}{c} P_{e}^{A}=q_{0}Q\left(\frac{\log\left(q_{0}/q_{1}\right)-E\left(\Lambda_{k}^{A}|\theta_{0}\right)}{\sqrt{\left[E\left[{\left(\Lambda_{k}^{A}\right)}^{2}|\theta_{0}\right]-E^{2}\left(\Lambda_{k}^{A}|\theta_{0}\right)\right]/K}}\right)\\ \;\;\;\;\;\;\;\;\;\;\;\;\;\;\;\;\;\;\;\;+q_{1}\left[1-Q\left(\frac{\log\left(q_{0}/q_{1}\right)-E\left(\Lambda_{k}^{A}|\theta_{1}\right)}{\sqrt{\left[E\left[{\left(\Lambda_{k}^{A}\right)}^{2}|\theta_{1}\right]-E^{2}\left(\Lambda_{k}^{A}|\theta_{1}\right)\right]/K}}\right)\right] \end{array}$$

### 4.2. Optimization of Comparison Thresholds

In Section 4.1, the asymptotic error probabilities at the AFC for extremely low and high SNR scenarios are obtained. They would be taken as the utility function for optimizing $t_{3},t_{2}$ and $t_{1}$ in this section. Our design target is to minimize the error probability of the AFC while satisfying the constraints of perfect secrecy and energy limitation. This problem can be stated as follows:

(30) $$\begin{array}{ccc} \;P0: & \min_{t_{3},t_{2},t_{1}}\;P_{e}^{A} & \\ & subject\;to: & \lambda_{1}=\lambda_{2}\\ & & \lambda_{1}+\lambda_{2}\leq \beta \end{array}$$

where the first constraint is the perfect secrecy condition to make the EFC totally be confused [10]. The second inequality constraint is to guarantee the specified energy efficiency.

Observing the Equations (23) and (29), we find that the numerical integration is included in $P_{e}^{A}$ and the variables to be optimized exist in the integral limits in a complicated form. These raise the difficulty to solve the problem. The utility function should be simplified.

Fortunately, it can be seen that $P_{e}^{A}$ decreases with $E(\Lambda_{k}^{A}|\theta_{1})$ and increases with $E(\Lambda_{k}^{A}|\theta_{0})$ since the impact of the variance of $\Lambda_{k}^{A}$ can be ignored compared with its mean for a large *K*. Therefore, $E\left(\Lambda_{k}^{A}|\theta_{1}\right)-E\left(\Lambda_{k}^{A}|\theta_{0}\right)$ can be used to replace the cost function in P0. The same idea was used in [9] to find the optimal encryption matrix. Thus, the optimization problem under the case of low SNR is given by

(31) $$\begin{array}{c} \;P1:\;\max_{t_{3},t_{2},t_{1}}\;\Gamma \left(\lambda^{*},t_{3},t_{2},t_{1}\right)\left[E\left(y_{k}^{A}|\theta_{1}\right)-E\left(y_{k}^{A}|\theta_{0}\right)\right]\\ \;\;subject\;to:\;\;\;\lambda_{1}=\lambda_{2}\\ \;\;\;\;\;\;\;\;\;\;\;\lambda_{1}+\lambda_{2}\leq \beta \end{array}$$

From Equations (19)∼(21), we achieve

(32) $$\Gamma \left(\lambda^{*},t_{3},t_{2},t_{1}\right)\left[E\left(y_{k}^{A}|\theta_{1}\right)-E\left(y_{k}^{A}|\theta_{0}\right)\right]=\frac{4{\left(P_{d}-P_{f}\right)}^{2}}{\delta_{A}^{2}}{\left[m\left(t_{1}\right)-n\left(t_{3},t_{2}\right)\right]}^{2}$$

Because the first item of the right side in Equation (32) is independent on the variables to be optimized, the final object is to maximize $\left|D(t_{3},t_{2},t_{1})\right|=\left|m\left(t_{1}\right)-n\left(t_{3},t_{2}\right)\right|$ while keep $\lambda_{1}=\lambda_{2}$ and $\lambda_{1}+\lambda_{2}\leq \beta$. Moreover, according to the Rayleigh distribution function, we have

(33) $$\lambda_{1}=exp\left(-t_{1}^{2}\right)\;\;\;\;\mathrm{and}\;\;\;\;\lambda_{2}=exp\left(-t_{3}^{2}\right)-exp\left(-t_{2}^{2}\right)$$

Now, in order to determine three appropriate thresholds, we should discuss the relationship of the target function $D(t_{3},t_{2},t_{1})$ and the actual energy consumption indicator, i.e., $\alpha =\lambda_{1}+\lambda_{2}$. Taking the $D(t_{3},t_{2},t_{1})$ as a function of *α*, we can derive that

(34) $$\delta_{D}\left(\alpha \right)=\frac{dD(\alpha )}{d\alpha }=\left\{\begin{array}{c} \frac{t_{1}-t_{2}}{2},\;\;\;\;\;\;\;\;\;\;\;\;\;\;\;\;\;\;\;\;\;t_{3}=0\\ t_{1}-t_{3},\;\;\;\;\;\;\;\;\;\;\;\;\;\;\;\;\;\;t_{1}=t_{2}\;\; \end{array}\right.$$

The detail of the calculation process for Equation (34) is shown in Appendix E.

From Equation (34), it can be easily seen that $\delta_{D}\left(\alpha \right)\geq 0$ for both cases of $t_{3}=0$ and $\;t_{1}=t_{2}$ due to the fact $0\leq t_{3}\leq t_{2}\leq t_{1}<\infty$. This results in that $D(t_{3},t_{2},t_{1})$ is strictly increasing with *α*. In particular, we can get $D(t_{3},t_{2},t_{1})=0$ for α=0. Thus, there is $D(t_{3},t_{2},t_{1})\geq 0$ at the whole range of α∈[0,1] and then the absolute calculation in the target function can be omitted. The above analysis contributes to that the equality (i.e., $\lambda_{1}+\lambda_{2}=\beta$) should be selected in the second constraint to maximize the cost function in Problem P1.

Moreover, we also find from Equation (34) that, with α→1, there is $\delta_{D}\left(\alpha \right)\to 0$ for $t_{3}=0$, while $\delta_{D}\left(\alpha \right)\to t_{1}$ for $t_{1}=t_{2}\;$. This finding further tells us $D(t_{3},t_{2},t_{1})$ will decrease faster for $t_{1}=t_{2}\;$ than for $t_{3}=0$ when *α* reduces from 1. Then, from the view of network robustness, choosing $t_{3}=0$ is preferred and this result will also be confirmed by the simulations given in Section 6.

Summarizing the above analysis can directly obtain the optimized thresholds given by

(35) $$t_{1}=\sqrt{\log(2/\beta )},\;\;\;\;\;\;\;t_{2}=\sqrt{\log[2/(2-\beta )]},\;\;\;\;t_{3}=0$$

Now, let’s come to the case of high SNR. Referring to the analyzing methods for the low SNR, the following optimization problem is established

(36) $$\begin{array}{c} \;P2:\;\max_{t_{3},t_{2},t_{1}}\;E\left(\Lambda_{k}^{A}|\theta_{1}\right)-E\left(\Lambda_{k}^{A}|\theta_{0}\right)\\ \;\;subject\;to:\;\;\;\lambda_{1}=\lambda_{2}\\ \;\;\;\;\;\;\;\;\;\;\;\lambda_{1}+\lambda_{2}\leq \beta \end{array}$$

Applying Equation (27) yields

(37) $$E\left(\Lambda_{k}^{A}|\theta_{1}\right)-E\left(\Lambda_{k}^{A}|\theta_{0}\right)=\left(\lambda_{1}+\lambda_{2}\right)\left(P_{d}-P_{f}\right)\log\frac{P_{d}\left(1-P_{f}\right)}{P_{f}\left(1-P_{d}\right)}$$

Obviously, the cost function is strictly increasing with $\lambda_{1}+\lambda_{2}$, since the local detection probability is always larger than the false alarm probability in practice so the item $\left(P_{d}-P_{f}\right)\log\frac{P_{d}\left(1-P_{f}\right)}{P_{f}\left(1-P_{d}\right)}$ is larger than zero. Thus, we should also choose $\lambda_{1}+\lambda_{2}=\beta$. However, which is better between $t_{1}=t_{2}\;$ and $t_{3}=0$ could not be determined from Equation (37). Actually, they have the identical detection performances for the extreme case of $\delta_{A}^{2}=0$. This phenomenon will be demonstrated in our simulations. Consequently, the thresholds given in Equation (35) should also be used under the high SNR situation.

## 5. Joint Local Decision and Wireless Transmission Based Secure Detection under Energy Constraint

In TCBO secure detection scheme, in order to meet the energy constraint of network, the sensors whose channel gains fall in the region between $t_{1}$ and $t_{2}$ (Consider the case of $t_{3}=0$.) will keep inactive. Of course, this gap between $t_{1}$ and $t_{2}$ can facilitate the AFC to tells the signals from flipping group and non-flipping group to some extent. However, the decision quality of the sensor’s local detection is not considered. That is to say the sensor with an error decision may be permitted to report its detection result to the FC, while the one with a correct decision perhaps is forbidden. We think this phenomenon maybe worsen the performance of decision fusion .

Therefore, we propose to select the dormant sensor basing on its local decision quality that can be quantified by the local Log-Likelihood Ratio $\Lambda_{k}^{L}$. Sensors with very small or very large LLR will send data to the fusion center, while the others stay silent to save energy. Obviously, this is the core idea of censoring sensor technique [8,11]. In particular, a perfectly secure distributed detection scheme with censoring sensors was given in [8]. But a comparatively ideal assumption was set that the EFC had no access to the data from sensors and only monitored the transmission activity of sensors. Moreover, the strategy in [8] did not consider the effect of the wireless transmission between the sensors and the fusion center on the reliability and security, so its applicability was limited. Basing on the above considerations, a joint local decision and wireless transmission based scheme for secure distributed detection with energy constraint is proposed in this section.

The JLDWT method is performed as follows: each sensor first calculates the local $\Lambda_{k}^{L}$ and compares it with two local decision thresholds. If $\Lambda_{k}^{L}$ locates between $\log(\lambda_{L}\cdot q_{0}/q_{1})$ and $\log(\lambda_{U}\cdot q_{0}/q_{1})$, it will stay inactive in current report timeslot for it appears less informative to make a correct decision about the target state. Otherwise, the sensor will make a 1bit-decision regarding the state of the hypothesis and then deliver it to the FC over a wireless PAC. While, in order to keep secret from the eavesdropping FC, the active sensor still should encrypt its local decision by randomly flipping it before transmitting. A single comparison threshold $t_{0}$ is used here instead of tree thresholds in TCBO scheme. If the sensor has the channel gain satisfying $\infty >h_{k}^{A}\geq t_{0}$, it is involved in the non-flipping group. Otherwise, it is chosen to be in the flipping group. At the fusion center, the LLR based fusion rule is still used. Three thresholds, namely $\log(\lambda_{L}\cdot q_{0}/q_{1})$, $\log(\lambda_{U}\cdot q_{0}/q_{1})$ and $t_{0}$, along with the encryption scheme at the sensors are assumed to be known by both the AFC and EFC.

### 5.1. Security Analysis

Now the condition of perfect secrecy in JLDWT scheme will be derived. Our analysis begins with the conditional likelihood function of the *k*-th sensor calculated by the EFC, which is given by

(38) $$\begin{array}{c} f\left(y_{k}^{E}|\theta_{i}\right)=\sum_{b_{k}}\sum_{x_{k}}\int_{0}^{\infty }f\left(y_{k}^{E},h_{k}^{A},x_{k},b_{k}|\theta_{i}\right)dh_{k}^{A}\\ =\sum_{b_{k}}\sum_{x_{k}}\int_{0}^{\infty }f\left(y_{k}^{E},h_{k}^{A},x_{k}|b_{k},\theta_{i}\right)p\left(b_{k}|\theta_{i}\right)dh_{k}^{A}\\ =\sum_{b_{k}}p\left(b_{k}|\theta_{i}\right)\sum_{x_{k}}\int_{0}^{\infty }f\left(y_{k}^{E}|h_{k}^{A},x_{k},b_{k},\theta_{i}\right)f\left(h_{k}^{A},x_{k}|b_{k},\theta_{i}\right)dh_{k}^{A}\\ \overset{\left(a\right)}{=}\sum_{b_{k}}p\left(b_{k}|\theta_{i}\right)\sum_{x_{k}}f\left(y_{k}^{E}|x_{k}\right)\int_{0}^{\infty }f\left(h_{k}^{A}\right)p\left(x_{k}|b_{k}\right)dh_{k}^{A}\\ \overset{\left(b\right)}{=}p\left(b_{k}=1|\theta_{i}\right)\left[f\left(y_{k}^{E}|x_{k}=1\right)\int_{t_{0}}^{+\infty }f\left(h_{k}^{A}\right)dh_{k}^{A}+f\left(y_{k}^{E}|x_{k}=-1\right)\int_{0}^{t_{0}}f\left(h_{k}^{A}\right)dh_{k}^{A}\right]\\ \;\;\;\;\;\;\;\;\;\;\;+p\left(b_{k}=0|\theta_{i}\right)\left[f\left(y_{k}^{E}|x_{k}=-1\right)\int_{t_{0}}^{+\infty }f\left(h_{k}^{A}\right)dh_{k}^{A}+f\left(y_{k}^{E}|x_{k}=1\right)\int_{0}^{t_{0}}f\left(h_{k}^{A}\right)dh_{k}^{A}\right]\\ \;\;\;\;\;\;\;\;\;\;\;+p\left(b_{k}=null|\theta_{i}\right)f\left(y_{k}^{E}|x_{k}=0\right)\int_{0}^{+\infty }f\left(h_{k}^{A}\right)dh_{k}^{A} \end{array}$$

where (a) is valid as $\theta_{i}\to b_{k}\to x_{k}\to y_{k}^{E}$ forms a Markov chain and $h_{k}^{A}$ is uncorrelated with $y_{k}^{E}$, $x_{k}$ and $\theta_{i}$. And (b) follows the fact that $p\left(x_{k}=1|b_{k}=1\right)=1$ and $p\left(x_{k}=-1|b_{k}=0\right)=1$ for $h_{k}^{A}\geq t_{0}$, while $p\left(x_{k}=-1|b_{k}=1\right)=1$ and $p\left(x_{k}=1|b_{k}=0\right)=1$ for $h_{k}^{A}<t_{0}$. In addition, $b_{k}=null$ corresponds to the sensor’s dormant state and $x_{k}=0$ accordingly. Furthermore, define $\lambda \overset{\mathrm{def}}{=}\int_{t_{0}}^{\infty }f\left(h_{k}^{A}\right)dh_{k}^{A}$ and we can easily yield

(39) $$\begin{array}{c} f\left(y_{k}^{E}|\theta_{1}\right)=P_{d}\left[f\left(y_{k}^{E}|x_{k}=1\right)\lambda +f\left(y_{k}^{E}|x_{k}=-1\right)\left(1-\lambda \right)\right]\\ \;\;\;\;\;\;\;\;\;\;\;\;\;\;\;\;\;\;\;\;\;\;\;+P_{m}\left[f\left(y_{k}^{E}|x_{k}=-1\right)\lambda +f\left(y_{k}^{E}|x_{k}=1\right)\left(1-\lambda \right)\right]+\left(1-P_{d}-P_{m}\right)f\left(y_{k}^{E}|x_{k}=0\right)\\ f\left(y_{k}^{E}|\theta_{0}\right)=P_{f}\left[f\left(y_{k}^{E}|x_{k}=1\right)\lambda +f\left(y_{k}^{E}|x_{k}=-1\right)\left(1-\lambda \right)\right]\\ \;\;\;\;\;\;\;\;\;\;\;\;\;\;\;\;\;\;\;\;\;\;\;+P_{0d}\left[f\left(y_{k}^{E}|x_{k}=-1\right)\lambda +f\left(y_{k}^{E}|x_{k}=1\right)\left(1-\lambda \right)\right]+\left(1-P_{f}-P_{0d}\right)f\left(y_{k}^{E}|x_{k}=0\right) \end{array}$$

To achieve perfect secrecy, two likelihood function $f\left(y_{k}^{E}|\theta_{1}\right)$ and $f\left(y_{k}^{E}|\theta_{0}\right)$ should be identical [10]. Then we can establish the following group of equations based on Equation (39).

(40) $$\begin{array}{c} \left(1-P_{d}-P_{m}\right)=\left(1-P_{f}-P_{0d}\right)\\ P_{d}\lambda +P_{m}\left(1-\lambda \right)=P_{f}\lambda +P_{0d}\left(1-\lambda \right)\\ P_{m}\lambda +P_{d}\left(1-\lambda \right)=P_{0d}\lambda +P_{f}\left(1-\lambda \right) \end{array}$$

Through simply computing, we obtain the perfect secrecy condition given by

(41) $$\lambda =1/2\;\;\;\;\;\;\mathrm{and}\;\;\;\;\;\;P_{d}+P_{m}=P_{f}+P_{0d}$$

The first condition in Equation (41) directly results in $t_{0}=\sqrt{\log(2)}$. And the second condition means that the activation probability under the hypothesis $\theta_{1}$, indicated by $\beta_{1}=P_{d}+P_{m}$, equates to the activation probability under $\theta_{0}$, denoted by $\beta_{2}=P_{f}+P_{0d}$. Comparing this condition with the perfect secrecy setting given in section II of [8], we find they are identical. Next, our task is to find two suitable thresholds $\lambda_{U}$ and $\lambda_{L}$ used in local Bayesian detection Equation (2) to minimize the error probability at the AFC, meanwhile, meet the perfect security and energy constraint of $\beta_{1}=\beta_{2}\leq \beta$.

### 5.2. Optimization of Local Detection Thresholds

Referring to the derivation methods of Equations (12) and (38), we can obtain the conditional likelihood functions at the AFC, which are expressed as

(42) $$\begin{array}{cc} f\left(y_{k}^{A}|\theta_{1}\right) & =P_{d}\left[\Phi \left(t_{0},\infty ,1,y_{k}^{A},\delta_{A}^{2}\right)+\Phi \left(0,t_{0},-1,y_{k}^{A},\delta_{A}^{2}\right)\right]\\ \;\;\;\;\;\;\; & +P_{m}\left[\Phi \left(t_{0},\infty ,-1,y_{k}^{A},\delta_{A}^{2}\right)+\Phi \left(0,t_{0},1,y_{k}^{A},\delta_{A}^{2}\right)\right]+\left(1-P_{d}-P_{m}\right)\Phi \left(0,\infty ,0,y_{k}^{A},\delta_{A}^{2}\right)\\ \;f\left(y_{k}^{A}|\theta_{0}\right) & =P_{f}\left[\Phi \left(t_{0},\infty ,1,y_{k}^{A},\delta_{A}^{2}\right)+\Phi \left(0,t_{0},-1,y_{k}^{A},\delta_{A}^{2}\right)\right]\\ \;\;\;\;\;\;\; & +P_{0d}\left[\Phi \left(t_{0},\infty ,-1,y_{k}^{A},\delta_{A}^{2}\right)+\Phi \left(0,t_{0},1,y_{k}^{A},\delta_{A}^{2}\right)\right]+\left(1-P_{f}-P_{0d}\right)\Phi \left(0,\infty ,0,y_{k}^{A},\delta_{A}^{2}\right)\;\;\;\; \end{array}$$

where $\Phi \left(t_{a},t_{b},x_{k},y_{k}^{A},\delta_{A}^{2}\right)$ has the expression of Equation (13).

#### 5.2.1. Optimization of Local Detection Thresholds under Low SNR

Following the deducing process in Section 4.1.1, we can obtain the calculation formula of the error probability under low SNR for AFC, which can be written as

(43) $$\begin{array}{c} P_{e}^{A}=q_{0}Q\left(\frac{\log\left(q_{0}/q_{1}\right)-\Gamma \left(\lambda_{U},\lambda_{L},t_{0}\right)E\left(y_{k}^{A}|\theta_{0}\right)}{\sqrt{\Gamma^{2}\left(\lambda_{U},\lambda_{L},t_{0}\right)\left[\delta_{A}^{2}-E^{2}\left(y_{k}^{A}|\theta_{0}\right)\right]/K}}\right)\\ \;\;\;\;\;\;\;\;\;\;\;\;\;\;\;\;\;\;\;\;+q_{1}\left[1-Q\left(\frac{\log\left(q_{0}/q_{1}\right)-\Gamma \left(\lambda_{U},\lambda_{L},t_{0}\right)E\left(y_{k}^{A}|\theta_{1}\right)}{\sqrt{\Gamma^{2}\left(\lambda_{U},\lambda_{L},t_{0}\right)\left[\delta_{A}^{2}-E^{2}\left(y_{k}^{A}|\theta_{1}\right)\right]/K}}\right)\right] \end{array}$$

where

(44) $$\Gamma \left(\lambda_{U},\lambda_{L},t_{0}\right)=\frac{\left(P_{d}-P_{f}\right)+\left(P_{0d}-P_{m}\right)}{\delta_{A}^{2}}\left[m\left(t_{0}\right)-n\left(0,t_{0}\right)\right]$$

In Equation (44), $P_{d}$, $P_{m}$, $P_{0d}$ and $P_{d}$ have the expressions given in Equation (7). Further, referring to the optimization problem P1, we build

(45) $$\begin{array}{c} \;P3:\;\max_{\lambda_{U},\lambda_{L}}\;\Gamma \left(\lambda_{U},\lambda_{L},t_{0}\right)\left[E\left(y_{k}^{A}|\theta_{1}\right)-E\left(y_{k}^{A}|\theta_{0}\right)\right]\\ \;\;subject\;to:\;\;\beta {\;}_{1}=\beta_{2}\leq \beta \end{array}$$

where

(46) $$\begin{array}{c} E\left(y_{k}^{A}|\theta_{1}\right)=\left(P_{d}-P_{m}\right)\left[m\left(t_{0}\right)-n\left(0,t_{0}\right)\right]\\ E\left(y_{k}^{A}|\theta_{0}\right)=\left(P_{f}-P_{0d}\right)\left[m\left(t_{0}\right)-n\left(0,t_{0}\right)\right] \end{array}$$

Applying Equation (46) to Equation (45), it can be achieved the rewritten object function is ${\left[\left(P_{d}-P_{f}\right)+\left(P_{0d}-P_{m}\right)\right]}^{2}\cdot$ ${\left[m\left(t_{0}\right)-n\left(0,t_{0}\right)\right]}^{2}/\delta_{A}^{2}$. Due to ${\left[m\left(t_{0}\right)-n\left(0,t_{0}\right)\right]}^{2}/\delta_{A}^{2}$ being independent on the variables to be optimized, the final target function can reduce to

(47) $$O\left(\beta_{1}\right)=\left(P_{d}-P_{f}\right)+\left(P_{0d}-P_{m}\right)$$

In addition, because the probability of correct detection is always larger than the incorrect one in practice, we have $O(\beta_{1})\geq 0$. Moreover, the condition $\beta {\;}_{1}=\beta_{2}$ contributes to $\left(P_{d}-P_{f}\right)=\left(P_{0d}-P_{m}\right)$, and then $O\left(\beta_{1}\right)=2\left(P_{d}-P_{f}\right)$.

First of all, we should find a good $\beta {\;}_{1}$ that meets the constraint in Equation (45) to maximize $O(\beta_{1})$. Combining Equations (7) and (47), we have

(48) $$O\left(\beta_{1}\right)=2\int_{\log[\lambda_{U}\left(\beta_{1}\right)\cdot q_{0}/q_{1}]}^{\infty }\left[f\left(\Lambda_{k}^{L}|\theta_{1}\right)-f\left(\Lambda_{k}^{L}|\theta_{0}\right)\right]d\Lambda_{k}^{L}$$

Let’s first focus on the following function:

(49) $$D\left(\lambda \right)\overset{\mathrm{Def}}{=}\int_{\log(\lambda q_{0}/q_{1})}^{\infty }\left[f\left(\Lambda_{k}^{L}|\theta_{1}\right)-f\left(\Lambda_{k}^{L}|\theta_{0}\right)\right]d\Lambda_{k}^{L}$$

Applying the condition $\left(P_{d}-P_{f}\right)=\left(P_{0d}-P_{m}\right)$, we can get the result of $D\left(\lambda_{U}\right)=D\left(\lambda_{L}\right)$, which is derived in detail in Appendix F. Substituting Equation (6) into Equation (49), it can be obtained

(50) $$\begin{array}{c} D\left(\lambda \right)=\frac{1}{\sqrt{2\pi snr_{L}}}\{\int_{\log(\lambda q_{0}/q_{1})}^{+\infty }\exp\left[-{\left(\Lambda_{k}^{L}-snr_{L}/2\right)}^{2}/\left(2snr_{L}\right)\right]d\Lambda_{k}^{L}\\ \;\;\;\;\;\;\;\;\;\;\;\;\;\;\;\;\;\;\;\;\;\;\;\;\;\;\;\;\;\;\;\;\;\;\;\;\;\;\;\;\;\;\;\;\;\;\;\;\;-\int_{\log(\lambda q_{0}/q_{1})}^{+\infty }\exp\left[-{\left(\Lambda_{k}^{L}+snr_{L}/2\right)}^{2}/\left(2snr_{L}\right)\right]d\Lambda_{k}^{L}\}\\ \;\;\;\;\;\;\;\;\;\;\;\;\;\;\;\;\;\;\;\;\;\;\;\;\;\;=\frac{1}{2}\left\{\mathrm{erf}\left(\left[\log\left(\lambda \frac{q_{0}}{q_{1}}\right)+snr_{L}/2\right]/\sqrt{2snr_{L}}\right)-\mathrm{erf}\left(\left[\log\left(\lambda \frac{q_{0}}{q_{1}}\right)-snr_{L}/2\right]/\sqrt{2snr_{L}}\right)\right\} \end{array}$$

where the error function $\mathrm{erf}\left(x\right)=\frac{2}{\sqrt{\pi }}\int_{0}^{x}\exp(-\eta^{2})d\eta$. Due to $\mathrm{er}f^{\prime }\left(x\right)=\frac{2}{\sqrt{\pi }}\exp\left(-x^{2}\right)$, we further get

(51) $$\begin{array}{c} \frac{dD\left(\lambda \right)}{d\lambda }=\frac{1}{\lambda \sqrt{2\pi snr_{L}}}(\exp\left\{-{\left[\log\left(\lambda q_{0}/q_{1}\right)+snr_{L}/2\right]}^{2}/2snr_{L}\right\}\\ \;\;\;\;\;\;\;\;\;\;\;\;\;\;\;\;\;\;\;\;\;\;\;\;\;\;\;\;\;\;\;\;\;\;\;\;\;\;\;\;\;\;\;\;\;\;\;\;\;\;\;\;\;\;\;\;\;\;\;\;\;\;\;\;\;\;\;\;\;\;\;\;\;\;\;\;\;\;\;\;\;\;\;\;\;\;\;\;\;\;\;\;\;\;\;\;-\exp\{-{\left[\log\left(\lambda q_{0}/q_{1}\right)-snr_{L}/2\right]}^{2}/2snr_{L}\}) \end{array}$$

Through setting $\frac{dD\left(\lambda \right)}{d\lambda }=0$, we can find three extreme points

(52) $$\lambda =0,\;\;\;\;\;\lambda =\infty \;\;\;\;\mathrm{and}\;\;\;\;\lambda =q_{1}/q_{0}$$

Substituting them into Equation (50), we have

(53) $$D\left(\lambda =0\right)=0,\;\;\;D\left(\lambda =\infty \right)=0\;\;\;\;\;\mathrm{and}\;\;\;\;\;D\left(\lambda =q_{1}/q_{0}\right)=\mathrm{erf}\left(\sqrt{\frac{snr_{L}}{8}}\right)$$

**Figure 3:**
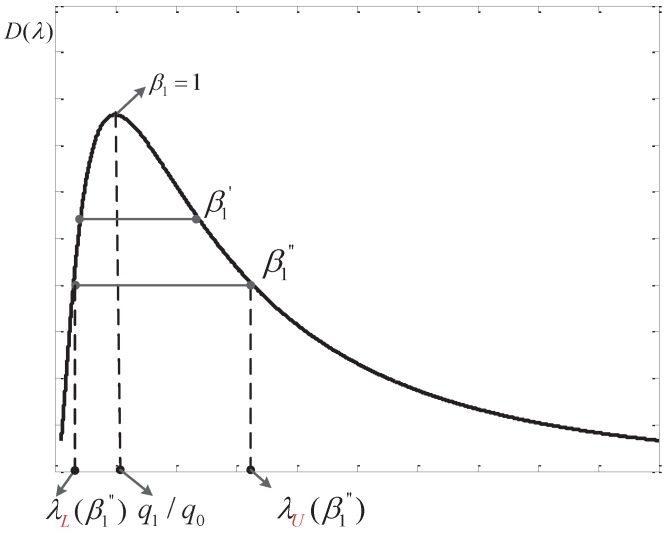
Diagram of the function $D\left(\lambda \right)$.

Based on Equation (53), we can draw a notional curve of $D\left(\lambda \right)$ as in Figure 3.

From Figure 3, we can see that there are two thresholds corresponding to one value of $D\left(\lambda \right)$, further, this $D\left(\lambda \right)$ actually maps to a single $\beta_{1}$. When $\beta_{1}=1$, two thresholds overlap at a point of $\lambda =q_{1}/q_{0}$ and $D\left(\lambda \right)$ has the maximum value. While $\beta_{1}$ decreases, we know that $\lambda_{U}$ moves towards *∞* and $\lambda_{L}$ approaches zero further. Thus, from Figure 3, we can see that the corresponding $D\left(\lambda \right)$ reduces. That is to say a larger $\beta_{1}$ is preferred in order to get a higher $D\left(\lambda \right)$.

Moreover, the reduced target function for P3 can be written as $O\left(\beta_{1}\right)=2D\left(\lambda_{U}\left(\beta_{1}\right)\right)$. Therefore, $\beta_{1}=\beta$ should be chosen to achieve the maximum $O(\beta_{1})$, along with the optimal performance of AFC, and the corresponding pair of thresholds are the optimal thresholds to be found. However, the expressions in Equations (7) and (49) are so complex that a closed-form expression of $\lambda_{L}\left(\beta \right)$ and $\lambda_{U}\left(\beta \right)$ couldn’t be obtained. In this situation, a pre-calculated table corresponding to each $snr_{L}$ could be used to get the required $\lambda_{L}\left(\beta \right)$ and $\lambda_{U}\left(\beta \right)$, just as the processing method in our simulations.

#### 5.2.2. Optimization of Local Detection Thresholds under High SNR

For the very high SNR scenario, the analysis methods in Section 4.1.2 are consulted. Firstly, the simplified LLR similar as Equation (26) are obtained, which is given by

(54) $$\Lambda_{k}^{A}=\left\{\begin{array}{c} 0,\;\;\;\;\;\;\;\;\;\;\;\;\;\;\;\;\;\;\;\;\;\;\;\;\;\;\;\;\;\;\;\;\;\;\;\;y_{k}^{A}=0\\ \log\frac{P_{d}}{P_{f}},\;\;\;\;\;\;\;\;{\hat{h}}_{k}^{A}\geq t_{h}\cap y_{k}^{A}>0\\ \log\frac{P_{m}}{P_{0d}},{\hat{h}}_{k}^{A}\geq t_{h}\cap y_{k}^{A}<0\\ \log\frac{P_{m}}{P_{0d}},{\hat{h}}_{k}^{A}<t_{h}\cap y_{k}^{A}>0\\ \log\frac{P_{d}}{P_{f}},\;\;\;\;\;\;\;\;{\hat{h}}_{k}^{A}<t_{h}\cap y_{k}^{A}<0 \end{array}\right.\;\;\;$$

where $t_{h}$ is set as $t_{0}$. Referring to the derivation of Equation (27), it is achieved that

(55) $$\begin{array}{c} E\left(\Lambda_{k}^{A}|\theta_{1}\right)=P_{d}\log\frac{P_{d}}{P_{f}}+P_{m}\log\frac{P_{m}}{P_{0d}}\\ E\left(\Lambda_{k}^{A}|\theta_{0}\right)=P_{f}\log\frac{P_{d}}{P_{f}}+P_{0d}\log\frac{P_{m}}{P_{0d}} \end{array}$$

Then the design problem is built as

(56) $$\begin{array}{c} \; \end{array}P4:\;\max_{\lambda_{u},\lambda_{l}}\;E\left(\Lambda_{k}^{A}|\theta_{1}\right)-E\left(\Lambda_{k}^{A}|\theta_{0}\right)\;\;subject\;to:\;\;\;\;\;\;\;\;\;\;\;\;\beta {\;}_{1}=\beta_{2}\leq \beta$$

Here, the object function can be written as $O\left(\lambda_{L},\lambda_{U}\right)=\left(P_{d}-P_{f}\right)\log\frac{P_{d}}{P_{f}}\cdot \log\frac{P_{0d}}{P_{m}}$. Because $P_{d}-P_{f}=P_{0d}-P_{m}$, maximizing $P_{d}-P_{f}$ could also make $\log\frac{P_{d}}{P_{f}}$ and $\log\frac{P_{0d}}{P_{m}}$ largest. Therefore, the object function in Equation (56) can be transformed into $P_{d}-P_{f}$, so Problem P4 is equivalent to Problem P3 and they have the identical optimization results.

## 6. Simulation Results and Discussions

In this section, simulation results are presented to evaluate the TCBO and the proposed JLDWT schemes in a sensor network of IoT. Their error probabilities are compared from various perfectives, including with the changing of transmission channel SNR, energy constraint and local detection SNR. The performance of a degraded form of the JLDWT scheme, where the random flipping is not included, is also given to represent the performance of secure detection designed in [8] over a practical rather than an idea wireless PAC.

### 6.1. Simulation Settings

A wireless sensor network with *K* sensors is modeled. The local detection SNR and the transmission channel SNR to fusion center for different sensors are assumed to be identical, as well as, the transmission channel SNR to the AFC and the EFC is also supposed to be equal. In addition, the LLR computation at the EFC is same as the AFC except the received signals from the sensors. Detail simulation parameters are listed in Table 1. Moreover, Table 2 and Table 3 give the specific local decision thresholds corresponding to different energy constraints under $snr_{L}=0\;\mathrm{dB}$ and $snr_{L}=5\;\mathrm{dB}$, respectively.

### 6.2. Simulation Results for TCBO Scheme

**Figure 4:**
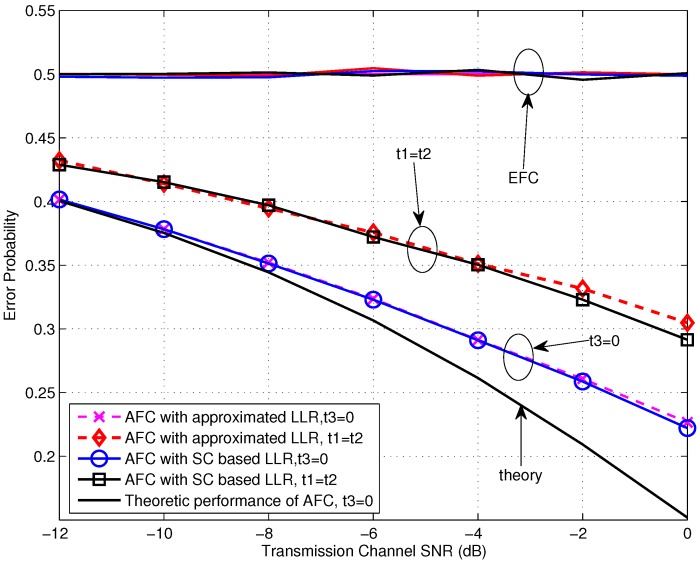
Error probabilities at the AFC and EFC as functions of various SNR for β=0.8 and $snr_{L}=5\;\;$ dB over low SNR region.

Let’s begin with the performance evaluation for the low SNR scenarios, where the SNR is not larger than 0 dB. From Figure 4, we first notice that the error probabilities for various settings at the EFC all locate around 0.5, which is our expected situation of perfect secrecy. Moreover, it is obvious that the AFC performance for the case of $t_{3}=0$ expresses better than the case of $t_{1}=t_{2}$ and there is a gain of about 4 dB obtained by the former one. This may be contributed by two aspects. On one side, the dormant region (or a gap) locates between the flipping and non-flipping group for the case of $t_{3}=0$ and it is beneficial for the AFC to discriminate between the flipping and non-flipping case, especially with serious noise. On the other side, the flipped decisions also disturb the fusion process at the AFC. For $t_{3}=0$, the power of received interference is lower since the flipping sensor has the lower channel gain. Thus the interference would have less effect on the fusion decision of the AFC. In addition, the error performances of using the approximated LLR (given in Equation (19)) are almost identical with the ones of using the statistic channel (SC) based LLR (Here, numerical integrations are needed.), particularly during the very low SNR region. This demonstrates the availability of the approximated LLR under low SNR. The theoretic performance calculated by using Equation (23) for $t_{3}=0$ is also drawn in Figure 4. It can be seen that the simulation result fits the theoretic one well for the SNR lower than −10 dB, and the gap between them becomes larger with the growing of SNR due to the noise variance being farther from the assumption of $\delta_{A}^{2}=\infty$ included in Equation (23).

**Figure 5:**
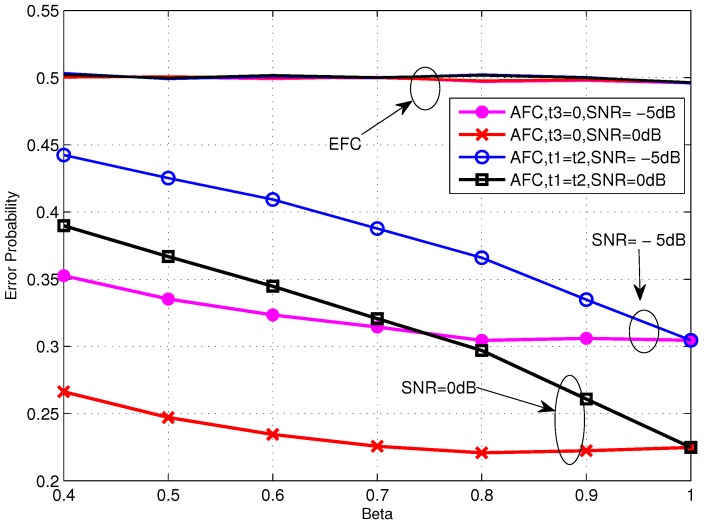
Error probabilities at the AFC and EFC as functions of various *β* for $snr_{L}=5\;\;$ dB over low SNR region.

Figure 5 shows the performance of TCBO scheme with the SC based LLR varying with *β*. It can be seen that the error probabilities for $t_{3}=0$ and $t_{1}=t_{2}$ are identical with β=1 and they would increase with *β* reducing from 1. But the increasing of the former one is slower than the latter one, which is correspondent to the analysis about Equation (34) in Section 4.2. Moreover, carefully observing the curves corresponding to $t_{3}=0$, we find that the error probabilities even rise slowly when we continue improving *β* and this phenomenon is more obvious for the moderate SNR, for example SNR=0 dB. It is because the reduced gap between the flipping and non-flipping group with a larger *β* leads to the confusion of the AFC to judge between two groups. It is noted that the confusion is created by the noise of channels. When the noise is very strong (or there is no such gap and the case $t_{1}=t_{2}$ follows this situation), the confusion always exits, so the more energy consumes and the better performance gets, just as the analytical result under low SNR in Section 4.2. However, with the noise reducing, the confusion disappears when the gap is large (Corresponding to a small *β*), while it appears when the gap becomes small. Therefore, although the energy consumption increases with *β* becoming large, the appeared confusion would worsen the performance. Of course, when the noise reduces to zero, the confusion never appears and the error probability will strictly decrease with *β*. This is the asymptotical analysis result under high SNR in Section 4.2 and also will be seen in the following simulations.

**Figure 6:**
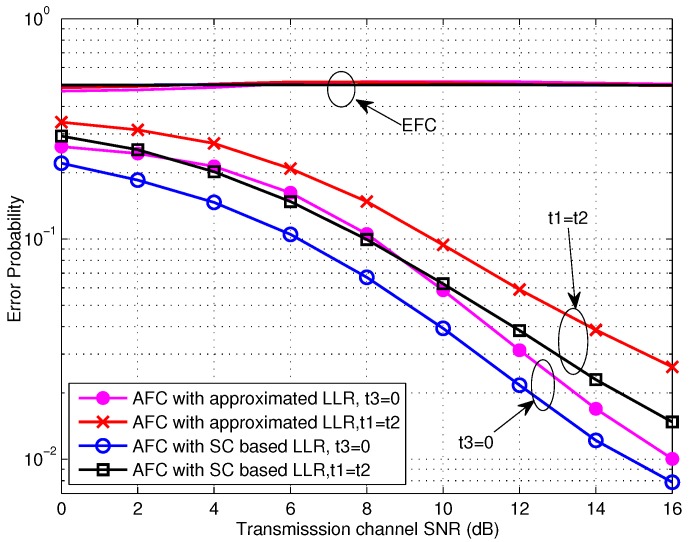
Error probabilities at the AFC and EFC as functions of various SNR for β=0.8 and $snr_{L}=5\;\;$ dB over high SNR region.

**Figure 7:**
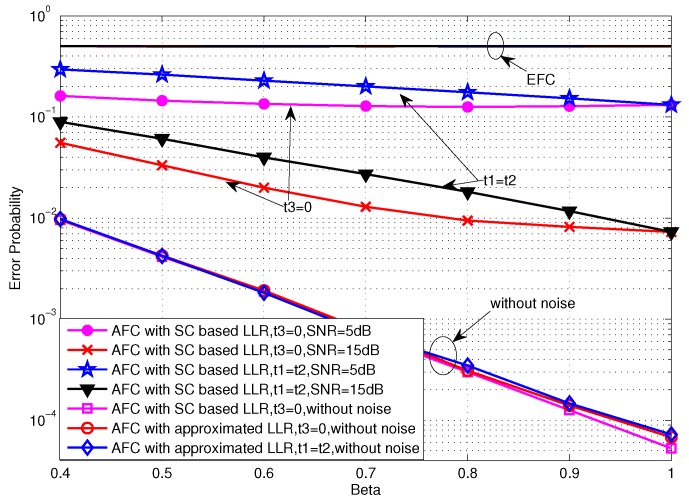
Error probabilities at the AFC and EFC as functions of various *β* for $snr_{L}=5\;\;$ dB over high SNR region.

The performance curves of TCBO scheme for the high SNR scenarios, where the SNR is larger than 0 dB, are shown in Figure 6. Obviously, the error probabilities for various simulation conditions at the EFC are all about 0.5 and perfect secrecy is maintained. Moreover, the AFC performance for the case of $t_{3}=0$ is still better than the one for the case of $t_{1}=t_{2}$, and the performance gap is about 2 dB. However, we find that the performance loss induced by the approximation of LLR with $\delta_{A}^{2}\to 0$ (seen in Equation (26)) is obvious. And this loss for $t_{3}=0$ will decrease with improving SNR, since the noise variance is closer to zero. In fact, the performance loss for $t_{1}=t_{2}$ will also reduce with the growing of SNR. In particular, this loss will reduce to zero for the extreme case of $\delta_{A}^{2}=0$ with two kinds of threshold setting, which can be seen in Figure 7. Therefore, the approximated LLR given in Equation (26) is still usable in terms of the reducing computation complexity, especially under high SNR scenarios.

From the other perspective, Figure 7 draws the error performance varying with *β* under the high SNR case. It can be seen that the threshold setting of $t_{3}=0$ demonstrates higher robustness than $t_{1}=t_{2}$ when the energy constraint is more severe. In addition, for the extreme case that the noise disappears, the error probabilities for various settings converge to an identical value and they decrease strictly as *β* increases. Because the confusion of the AFC for discriminating between the flipping and non-flipping group does not exist when the noise is absent, the case of $t_{3}=0$ would be equivalent to the case of $t_{1}=t_{2}$. Furthermore, the approximated LLR could obtain the similar performance as the SC based LLR.

### 6.3. Simulation Results for JLDWT Scheme

**Figure 8:**
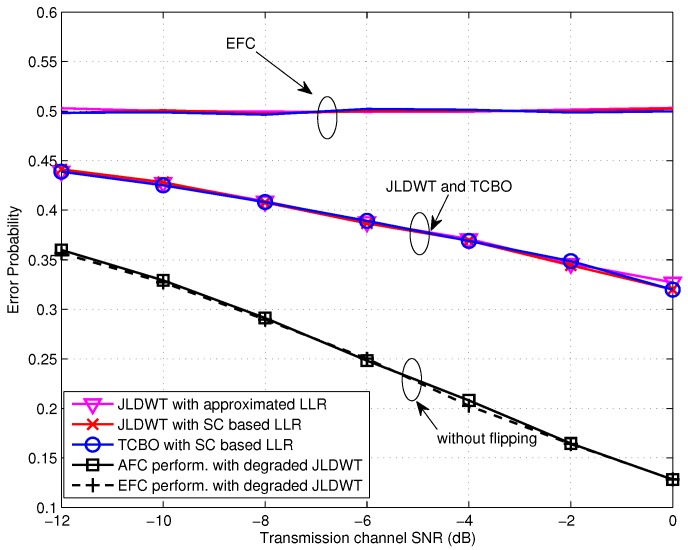
Error probabilities of TCBO and JLDWT schemes as functions of various SNR for β=0.8 and $snr_{L}=0\;\;$ dB over low SNR region.

In this section, the performances of the TCBO and the proposed JLDWT schemes are compared from various perspectives. Figure 8 gives the error probabilities of two schemes for low SNR case. We can see that the JLDWT using the SC based LLR, the JLDWT using the approximated LLR and the TCBO using the SC based LLR have almost identical performance. Because the strong channel noise dominates in low SNR, the JLDWT’s advantage is not shown up. The simplified LLR for low SNR is very effective for maintaining the performance as well as reducing complexity of FC. Furthermore, all these schemes could achieve the perfect secrecy.

For comparison, the degraded JLDWT method without random flipping is also evaluated. Concretely, in the degraded JLDWT scheme, each sensor still executes the local detection based on the Bayesian criteria with two local decision thresholds $\lambda_{U}$ and $\lambda_{L}$ keeping $\beta_{1}=\beta_{2}$, while the active sensor will deliver the local 1bit-decision in its original form to the FC no matter what the estimated channel gain is. That is to say the difference of the degraded JLDWT from the JLDWT is that the flipping process is not involved. As comparing it with the secure strategy given in [8], we can easily see that $\lambda_{U}$ and $\lambda_{L}$ used by the degraded JLDWT are identical with the ones used by the scheme in [8], because their optimization targets to find the optimal $\lambda_{U}$ and $\lambda_{L}$ are equivalent and the perfect secrecy constraint conditions are same. Thus, the degraded JLDWT can be seen equivalent to the scheme of [8] except that it is applied under a more realistic scenario considering the wireless transmission and a looser constraint on the EFC ability relative to the case in [8]. From Figure 8, it can be seen that the secrecy from the EFC is totally lost and the EFC has the same performance as the AFC when the secure strategy in [8] is used. That is to say the strategy given in [8] is ineffective if the EFC has the same process capability and the prior information as the AFC. Thus, random flipping is necessary to assure the information confidentiality with the enhanced EFC. Certainly, this information security is exchanged by certain performance loss of the AFC.

**Figure 9:**
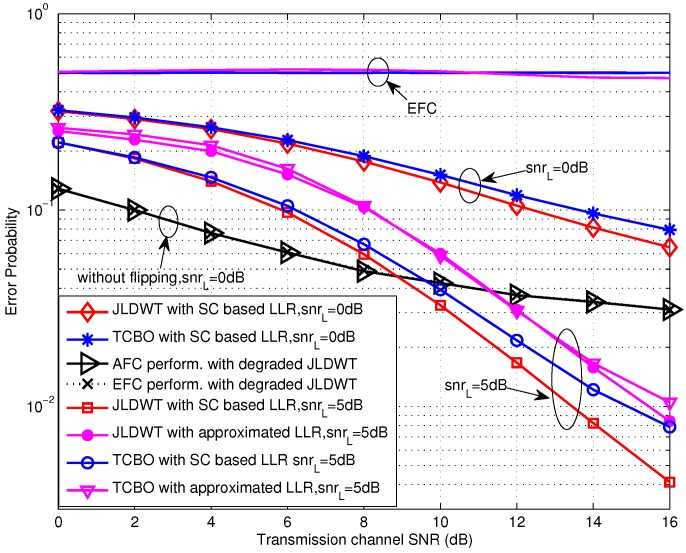
Error probabilities of TCBO and JLDWT schemes as functions of various SNR for β=0.8 over high SNR region.

As for the case of high SNR, it can be seen from Figure 9 that the JLDWT scheme with the SC based LLR outperforms the TCBO using the SC based LLR and the performance gain for the AFC would increase with the transmission channel SNR going higher. That is to say preventing the worse local decision from contributing to the data fusion would facilitate to improve the performance at the FC when the disadvantage effect of transmission channel reduces. Moreover, similar as the result seen in Figure 8, the AFC and the EFC have the identical error probabilities with the degraded JLDWT and the information confidentiality is not guaranteed. In addition, the approximated LLR contributes to the performance loss for both the JLDWT and the TCBO schemes, but the JLDWT scheme still outperforms the TCBO one slightly.

**Figure 10:**
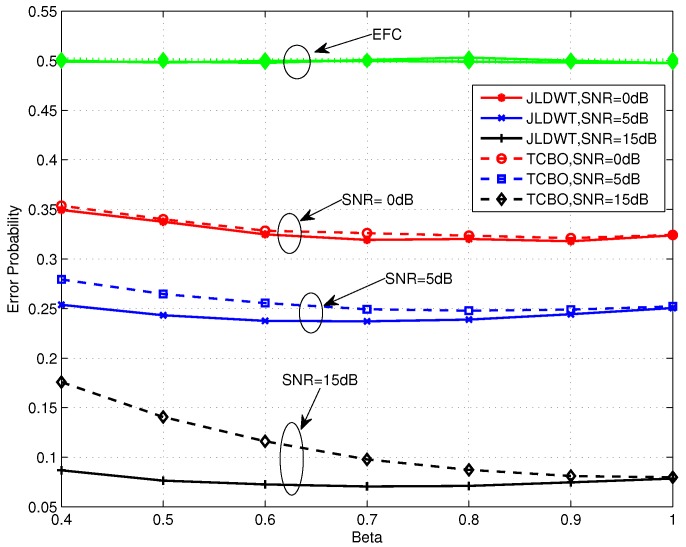
Error probabilities of TCBO and JLDWT schemes with SC based LLR as functions of various *β* for $snr_{L}=0\;\;$dB.

**Figure 11:**
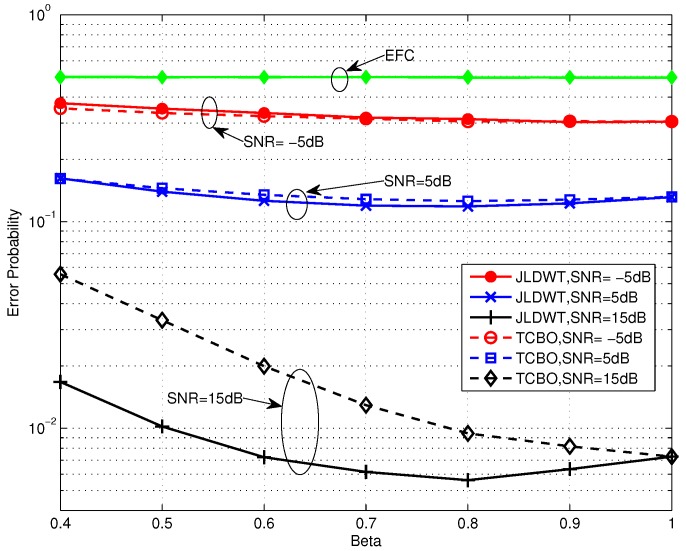
Error probabilities of TCBO and JLDWT schemes with SC based LLR as functions of various *β* for $snr_{L}=5\;\;$dB.

Figure 10 and Figure 11 compare the error performance of TCBO and JLDWT schemes with the SC based LLR under $snr_{L}=0\;\;\mathrm{dB}$ and $snr_{L}=5\;\;\mathrm{dB}$, respectively. It can be seen that the gain of the JLDWT scheme against the TCBO method increases with the growing of transmission channel SNR. That is correspondent to the result seen in Figure 9. Furthermore, this gain at the high SNR, for example SNR = 15 dB, becomes larger for a smaller *β*. That is the advantage induced by cancelling the worse local detection results from the fusion data and it would dominant the final decision fusion when the transmission channel becomes good. Furthermore, we also find the performance inflection phenomenon over the curves of the JLDWT, which is similar as seen in Figure 5. While, it is induced by the confusion of the sensor to judge between two hypothesis of $\theta_{0}$ and $\theta_{1}$, rather than the confusion of the AFC for discriminating between the flipping and non-flipping group.

Based on the above simulation results and discussions, we suggest that the TCBO scheme with the approximated LLR is a good selection over the low transmission channel SNR region. While, under a good wireless transmission scenario with a severe energy constraint, the JLDWT scheme with the SC based LLR is preferred in order to obtain the higher detection accuracy at the AFC. Moreover, a moderate *β* around 0.7∼0.8 is more appropriate for a practical sensor network in terms of both the energy consumption and the detection performance. In addition, it is to be noted that the TCBO and JLDWT schemes both can be easily extended to a larger sensor network, although only the case of *K* = 20 is studied in our simulations.

## 7. Conclusions and Future Work

Distributed detection scheme with good security and energy efficiency plays an important role in the implement of sensor network in IoT. In this paper, two secure decentralized detection schemes under energy constraint are studied comprehensively. Firstly, a specific energy constraint is introduced to the existing channel aware encryption scheme and we call it TCBO scheme. Next, the simplified LLRs under the low and high SNR are derived, respectively. Based on the new LLRs, the asymptotic error probabilities for the worst and best noise situations at the AFC are calculated. Then, three comparison thresholds are optimized through minimizing the error probability while satisfying the perfect secrecy and energy constraints. Secondly, combing the local detection and the wireless transmission of local decision at the sensor, a hybrid scheme named JLDWT is proposed, where the energy efficiency is provided by censoring the sensor with less informative local LLR and the confidentiality from the EFC is guaranteed by the channel based random flipping. Then, the asymptotic error probabilities under low and high SNR environment are also given. Furthermore, two local detection thresholds and one flipping comparison threshold are optimized to minimize the error rates, as well as, assure the perfect secrecy and the required energy efficiency. At last, we evaluate the detection performance of the TCBO and the proposed JLDWT schemes through computer simulations. The simulation results demonstrate that the perfect secrecy is assured by both schemes. The JLDWT scheme outperforms the TCBO one under the better wireless transmission environment with a severe energy constraint.

The perfect secrecy is guaranteed at the cost of reducing the detection accuracy at the AFC in the TCBO and JLDWT schemes. However, in some scenarios, a limited information leakage to the EFC maybe is permitted, while the high detection performance at the AFC is more important. In future work, the modified forms of the above two schemes will be designed to support the more flexible constraint on the EFC’s performance. Moreover, except the eavesdropping attack, there are many other attack modes faced by IoT networks in practice, such as the denial of services (DOS) attack, node outage attack, signal jamming attack and intentional attack. Among them, the intentional attack could incur fatal threat on network by paralyzing a small fraction of nodes with highest degrees. As to IoT networks, if some important nodes, such as the fusion center and the controller, suffer the intentional attack, the whole IoT system may be disrupted. Therefore, the robust defense mechanism against the intentional attack for IoT will be studied in our future work.

## Figures and Tables

**Table 1:** Simulation Parameters in Wireless Sensor Network.

Parameters	Assumption
Number of sensors	20
Prior probabilities of target states	$q_{0}=q_{1}=0.5$
Transmission channel model	Rayleigh distribution with $E[h^{2}]=1$
Energy constraint	β=0.4:0.1:1
Local detection SNR	$snr_{L}=0,\;\;\;\;\;5\;\;\;$dB
Transmission channel SNR	$SNR_{A}=SNR_{E}=-12:2:16$ dB

**Table 2:** Two local decision thresholds $\lambda_{U}$ and $\lambda_{L}$ for $snr_{L}=0$ dB.

*β*	0.4	0.5	0.6	0.7	0.8	0.9	1
$\lambda_{U}$	2.585	2.145	1.810	1.545	1.330	1.155	1.000
$\lambda_{L}$	0.387	0.466	0.553	0.647	0.752	0.866	1.000

**Table 3:** Two local decision thresholds $\lambda_{U}$ and $\lambda_{L}$ for $snr_{L}=5$ dB.

*β*	0.4	0.5	0.6	0.7	0.8	0.9	1
$\lambda_{U}$	8.320	5.595	3.875	2.730	1.945	1.395	1.000
$\lambda_{L}$	0.120	0.179	0.258	0.366	0.514	0.717	1.000

## Appendix A. Approximation of Φ (*t_a_*, *t_b_*, *x_k_*, *y*${}_{k}^{A}$, *δ*${}_{A}^{2}$) under the Low Channel SNR

The derivation of the approximated formulation for $\Phi \left(t_{a},t_{b},x_{k},y_{k}^{A},\delta_{A}^{2}\right)$ is given by

(A1) $$\begin{array}{cc} \Phi \left(t_{a},t_{b},x_{k},y_{k}^{A},\delta_{A}^{2}\right) & =\int_{t_{a}}^{t_{b}}\frac{1}{\sqrt{2\pi }\delta_{A}}\exp\left(-\frac{{\left(y_{k}^{A}-h_{k}^{A}x_{k}\right)}^{2}}{2\delta_{A}^{2}}\right)2h_{k}^{A}\exp\left[-{\left(h_{k}^{A}\right)}^{2}\right]dh_{k}^{A}\\ & =N\left(y_{k}^{A},\delta_{A}^{2}\right)\int_{t_{a}}^{t_{b}}\exp\left(\frac{y_{k}^{A}h_{k}^{A}x_{k}}{\delta_{A}^{2}}\right)2h_{k}^{A}\exp\left[-\left(1+\frac{{x_{k}}^{2}}{2\delta_{A}^{2}}\right){\left(h_{k}^{A}\right)}^{2}\right]dh_{k}^{A}\\ & \overset{\left(a\right)}{\approx }N\left(y_{k}^{A},\delta_{A}^{2}\right)\int_{t_{a}}^{t_{b}}\left(1+\frac{y_{k}^{A}h_{k}^{A}x_{k}}{\delta_{A}^{2}}\right)2h_{k}^{A}\exp\left[-\left(1+\frac{{x_{k}}^{2}}{2\delta_{A}^{2}}\right){\left(h_{k}^{A}\right)}^{2}\right]dh_{k}^{A}\\ & =N\left(y_{k}^{A},\delta_{A}^{2}\right)\{-{\left(1+\frac{{x_{k}}^{2}}{2\delta_{A}^{2}}\right)}^{-1}\exp\left[-\left(1+\frac{{x_{k}}^{2}}{2\delta_{A}^{2}}\right){\left(h_{k}^{A}\right)}^{2}\right]\;\;{|}_{t_{a}}^{t_{b}}\\ & \;\;\;\;\;\;\;\;\;\;\;\;\;\;\;\;\;\;\;\;\;\;\;\;\;\;\;\;\;\;\;\;\;\;\;\;\;\;\;\;\;\;\;\;\;\;\;\;\;\;\;\;\;\;\;\;\;\;\;\;+\frac{y_{k}^{A}x_{k}}{\delta_{A}^{2}}\int_{t_{a}}^{t_{b}}2{\left(h_{k}^{A}\right)}^{2}\exp\left[-\left(1+\frac{{x_{k}}^{2}}{2\delta_{A}^{2}}\right){\left(h_{k}^{A}\right)}^{2}\right]dh_{k}^{A}\}\\ & \overset{\left(b\right)}{\approx }N\left(y_{k}^{A},\delta_{A}^{2}\right)\left\{exp\left(-t_{a}^{2}\right)-exp\left(-t_{b}^{2}\right)-\frac{y_{k}^{A}x_{k}}{\delta_{A}^{2}}\int_{t_{a}}^{t_{b}}h_{k}^{A}d\left(\exp[-{\left(h_{k}^{A}\right)}^{2}]\right)\right\}\\ & =N\left(y_{k}^{A},\delta_{A}^{2}\right)\{exp\left(-t_{a}^{2}\right)-exp\left(-t_{b}^{2}\right)\\ & \;\;\;\;\;\;\;\;\;\;\;\;\;\;\;\;\;\;\;\;\;\;\;\;\;\;\;\;\;\;\;\;\;\;\;\;\;\;\;\;\;\;\;\;\;\;\;\;\;\;\;\;\;\;\;\;\;\;\;\;+\frac{y_{k}^{A}x_{k}}{\delta_{A}^{2}}\left[t_{a}exp\left(-t_{a}^{2}\right)-t_{b}exp\left(-t_{b}^{2}\right)+\int_{t_{a}}^{t_{b}}exp(-h^{2})dh\right]\} \end{array}$$

where (a) is based on the fact that exp(x)≈1+x for small x and (b) is due to the assumption of $\delta_{A}^{2}\to \infty$.

## Appendix B. Calculation of Three Integrations Used in Equations (20) and (22)

(B1) $$\begin{array}{cc} \int_{-\infty }^{+\infty }y_{k}^{A}N\left(y_{k}^{A},\delta_{A}^{2}\right)dy_{k}^{A} & =\frac{1}{\sqrt{2\pi }\delta_{A}}\int_{-\infty }^{+\infty }y_{k}^{A}\exp\left(-\frac{{\left(y_{k}^{A}\right)}^{2}}{2\delta_{A}^{2}}\right)dy_{k}^{A}\\ & =-\frac{\delta_{A}}{\sqrt{2\pi }}\int_{-\infty }^{+\infty }d\left[\exp\left(-\frac{{\left(y_{k}^{A}\right)}^{2}}{2\delta_{A}^{2}}\right)\right]=0 \end{array}$$

(B2) $$\begin{array}{c} \int_{-\infty }^{+\infty }{\left(y_{k}^{A}\right)}^{2}N\left(y_{k}^{A},\delta_{A}^{2}\right)dy_{k}^{A}\;=\frac{1}{\sqrt{2\pi }\delta_{A}}\int_{-\infty }^{+\infty }{\left(y_{k}^{A}\right)}^{2}\exp\left(-\frac{{\left(y_{k}^{A}\right)}^{2}}{2\delta_{A}^{2}}\right)dy_{k}^{A}\\ \;\;\;\;\;\;\;\;\;\;\;\;\;\;\;\;\;\;\;\;\;\;\;\;\;\;\;\;\;\;\;\;\;\;\;\;\;\;\;\;\;\;\;\;\;\;\;\;\;\;\;\;\;\;\;\;\;\;\;\;\;\;=-\frac{\delta_{A}}{\sqrt{2\pi }}\int_{-\infty }^{+\infty }y_{k}^{A}d\left[\exp\left(-\frac{{\left(y_{k}^{A}\right)}^{2}}{2\delta_{A}^{2}}\right)\right]\\ \;\;\;\;\;\;\;\;\;\;\;\;\;\;\;\;\;\;\;\;\;\;\;\;\;\;\;\;\;\;\;\;\;\;\;\;\;\;\;\;\;\;\;\;\;\;\;\;\;\;\;\;\;\;\;\;\;\;\;\;\;\;=\frac{\delta_{A}}{\sqrt{2\pi }}y_{k}^{A}\exp\left[-\frac{{\left(y_{k}^{A}\right)}^{2}}{2\delta_{A}^{2}}\right]\left|\begin{array}{c} +\infty \\ -\infty \end{array}\right.+\frac{\delta_{A}}{\sqrt{2\pi }}\int_{-\infty }^{+\infty }\exp\left(-\frac{{\left(y_{k}^{A}\right)}^{2}}{2\delta_{A}^{2}}\right)dy_{k}^{A}\\ \;\;\;\;\;\;\;\;\;\;\;\;\;\;\;\;\;\;\;\;\;\;\;\;\;\;\;\;\;\;\;\;\;\;\;\;\;\;\;\;\;\;\;\;\;\;\;\;\;\;\;\;\;\;\;\;\;\;\;\;\;\;=0+\frac{\delta_{A}^{2}}{\sqrt{2\pi }\delta_{A}}\int_{-\infty }^{+\infty }\exp\left(-\frac{{\left(y_{k}^{A}\right)}^{2}}{2\delta_{A}^{2}}\right)dy_{k}^{A}=\delta_{A}^{2} \end{array}$$

(B3) $$\begin{array}{c} \int_{-\infty }^{+\infty }{\left(y_{k}^{A}\right)}^{3}N\left(y_{k}^{A},\delta_{A}^{2}\right)dy_{k}^{A}=\frac{1}{\sqrt{2\pi }\delta_{A}}\int_{-\infty }^{+\infty }{\left(y_{k}^{A}\right)}^{2}y_{k}^{A}\exp\left(-\frac{{\left(y_{k}^{A}\right)}^{2}}{2\delta_{A}^{2}}\right)dy_{k}^{A}\\ \;\;\;\;\;\;\;\;\;\;\;\;\;\;\;\;\;\;\;\;\;=-\frac{\delta_{A}}{\sqrt{2\pi }}\int_{-\infty }^{+\infty }{\left(y_{k}^{A}\right)}^{2}d\left[\exp\left(-\frac{{\left(y_{k}^{A}\right)}^{2}}{2\delta_{A}^{2}}\right)\right]\\ \;\;\;\;\;\;\;\;\;\;\;\;\;\;\;\;\;\;\;\;\;=\frac{\delta_{A}}{\sqrt{2\pi }}{\left(y_{k}^{A}\right)}^{2}exp\left(-\frac{{\left(y_{k}^{A}\right)}^{2}}{2\delta_{A}^{2}}\right)\left|\begin{array}{c} -\infty \\ +\infty \end{array}\right.+\frac{2\delta_{A}}{\sqrt{2\pi }}\int_{-\infty }^{+\infty }y_{k}^{A}\exp\left(-\frac{{\left(y_{k}^{A}\right)}^{2}}{2\delta_{A}^{2}}\right)dy_{k}^{A}\;\;\;=0 \end{array}$$

## Appendix C. Derivation of $\Lambda_{k}^{A}$ under High SNR

In Equation (25), substituting $f\left(y_{k}^{A}|x_{k},{\hat{h}}_{k}^{A}\right)$ with $\frac{1}{\sqrt{2\pi }\delta_{A}}\exp\left[-\frac{{\left(y_{k}^{A}-{\hat{h}}_{k}^{A}x_{k}\right)}^{2}}{2\delta_{A}^{2}}\right]$ gives

(C1) $$\begin{array}{c} \Lambda_{k}^{A}=\log\frac{f\left(y_{k}^{A}|\theta_{1}\right)}{f\left(y_{k}^{A}|\theta_{0}\right)}=\left\{\begin{array}{c} \log\left[\frac{P_{d}\exp\left(2y_{k}^{A}{\hat{h}}_{k}^{A}/\delta_{A}^{2}\right)+\left(1-P_{d}\right)}{P_{f}\exp\left(2y_{k}^{A}{\hat{h}}_{k}^{A}/\delta_{A}^{2}\right)+\left(1-P_{f}\right)}\right],{\hat{h}}_{k}^{A}\geq t_{h}\\ \log\left[\frac{\left(1-P_{d}\right)\exp\left(2y_{k}^{A}{\hat{h}}_{k}^{A}/\delta_{A}^{2}\right)+P_{d}}{\left(1-P_{f}\right)\exp\left(2y_{k}^{A}{\hat{h}}_{k}^{A}/\delta_{A}^{2}\right)+P_{f}}\right],{\hat{h}}_{k}^{A}<t_{h} \end{array}\right. \end{array}$$

Furthermore, with $\delta_{A}^{2}\to 0$, we have $\exp\left(\frac{2y_{k}^{A}{\hat{h}}_{k}^{A}}{\sigma_{A}^{2}}\right)\to \infty$ for $y_{k}^{A}>0$, $\exp\left(\frac{2y_{k}^{A}{\hat{h}}_{k}^{A}}{\sigma_{A}^{2}}\right)\to 0$ for $y_{k}^{A}<0$ and $\Lambda_{k}^{A}=0$ for $y_{k}^{A}=0$. Substituting them into Equation (C1), we can rewrite it as the Equation (26).

## Appendix D. Derivation of Equations (27) and (28)

From Equation (26), we see that $\Lambda_{k}^{A}$ is a discrete random variable, so its mean can be given by $E\left(\Lambda_{k}^{A}|\theta_{i}\right)=\sum_{\Lambda_{k}^{A}}\Lambda_{k}^{A}f\left(\Lambda_{k}^{A}|\theta_{i}\right)$. Moreover, the non-negative channel coefficient makes that $y_{k}^{A}>0$ is equivalent to $x_{k}=1$, $y_{k}^{A}<0$ corresponds to $x_{k}=-1$ and $y_{k}^{A}=0$ means $x_{k}=0$. In addition, $h_{k}^{A}\approx {\hat{h}}_{k}^{A}$ holds for the large SNR scenario. Combining the above facts, we have

(D1) $$\begin{array}{c} E\left(\Lambda_{k}^{A}|\theta_{i}\right)=\sum_{\Lambda_{k}^{A}}\Lambda_{k}^{A}f\left(\Lambda_{k}^{A}|\theta_{i}\right)\\ =0\cdot p\left(x_{k}=0|\theta_{i}\right)+\log\frac{P_{d}}{P_{f}}\left[\int_{t_{h}}^{+\infty }f\left(h_{k}^{A},x_{k}=1|\theta_{i}\right)dh_{k}^{A}+\int_{0}^{t_{h}}f\left(h_{k}^{A},x_{k}=-1|\theta_{i}\right)dh_{k}^{A}\right]\\ \;\;\;+\log\frac{1-P_{d}}{1-P_{f}}\left[\int_{t_{h}}^{+\infty }f\left(h_{k}^{A},x_{k}=-1|\theta_{i}\right)dh_{k}^{A}+\int_{0}^{t_{h}}f\left(h_{k}^{A},x_{k}=1|\theta_{i}\right)dh_{k}^{A}\right]\\ =\log\frac{P_{d}}{P_{f}}\left[\int_{t_{h}}^{+\infty }f\left(h_{k}^{A}|x_{k}=1,\theta_{i}\right)p\left(x_{k}=1|\theta_{i}\right)dh_{k}^{A}\;+\int_{0}^{t_{h}}f\left(h_{k}^{A}|x_{k}=-1,\theta_{i}\right)p\left(x_{k}=-1|\theta_{i}\right)dh_{k}^{A}\right]\\ +\log\frac{1-P_{d}}{1-P_{f}}\left[\int_{t_{h}}^{+\infty }f\left(x_{k}^{A}|u_{k}=-1,\theta_{i}\right)p\left(x_{k}=-1|\theta_{i}\right)dh_{k}^{A}+\int_{0}^{t_{h}}f\left(h_{k}^{A}|x_{k}=1,\theta_{i}\right)p\left(x_{k}=1|\theta_{i}\right)dh_{k}^{A}\right]\\ \overset{\left(a\right)}{=}\log\frac{P_{d}}{P_{f}}[\int_{t_{h}}^{+\infty }f\left(h_{k}^{A}\right)\sum_{b_{k}}p\left(x_{k}=1|b_{k}\right)p\left(b_{k}|\theta_{i}\right)dh_{k}^{A}+\int_{0}^{t_{h}}f\left(h_{k}^{A}\right)\sum_{b_{k}}p\left(x_{k}=-1|b_{k}\right)p\left(b_{k}|\theta_{i}\right)dh_{k}^{A}]\\ \;\;\;\;\;\;\;\;\;\;\;\;+\log\frac{1-P_{d}}{1-P_{f}}[\int_{t_{h}}^{+\infty }f\left(h_{k}^{A}\right)\sum_{b_{k}}p\left(x_{k}=-1|b_{k}\right)p\left(b_{k}|\theta_{i}\right)dh_{k}^{A}\\ \;\;\;\;\;\;\;\;\;\;\;+\int_{0}^{t_{h}}f\left(h_{k}^{A}\right)\sum_{b_{k}}p\left(x_{k}=1|b_{k}\right)p\left(b_{k}|\theta_{i}\right)dh_{k}^{A}]\\ \overset{\left(b\right)}{=}\log\frac{P_{d}}{P_{f}}\left[\int_{t_{1}}^{+\infty }f\left(h_{k}^{A}\right)p\left(b_{k}=1|\theta_{i}\right)dh_{k}^{A}\;+\int_{t_{3}}^{t_{2}}f\left(h_{k}^{A}\right)p\left(b_{k}=1|\theta_{i}\right)dh_{k}^{A}\right]\\ \;\;\;\;\;\;\;\;\;\;\;\;+\log\frac{1-P_{d}}{1-P_{f}}[\int_{t_{1}}^{+\infty }f\left(h_{k}^{A}\right)p\left(b_{k}=0|\theta_{i}\right)dh_{k}^{A}+\int_{t_{3}}^{t_{2}}f\left(h_{k}^{A}\right)p\left(b_{k}=0|\theta_{i}\right)dh_{k}^{A}]\\ =\left[\log\frac{P_{d}}{P_{f}}p\left(b_{k}=1|\theta_{i}\right)+\log\frac{1-P_{d}}{1-P_{f}}p\left(b_{k}=0|\theta_{i}\right)\right]\left(\lambda_{1}+\lambda_{2}\right) \end{array}$$

where (a) follows the condition that $h_{k}^{A}$ is uncorrelated with $x_{k}$ and $\theta_{i}$, and (b) is due to Equation (24). Similarly, it can be obtained that

(D2) $$\begin{array}{c} E\left({\left(\Lambda_{k}^{A}\right)}^{2}|\theta_{i}\right)=\sum_{\Lambda_{k}^{A}}{\left(\Lambda_{k}^{A}\right)}^{2}f\left(\Lambda_{k}^{A}|\theta_{i}\right)\\ =0^{2}\cdot p\left(x_{k}=0|\theta_{i}\right)\;+{\left(\log\frac{P_{d}}{P_{f}}\right)}^{2}\left[\int_{t_{h}}^{+\infty }f\left(h_{k}^{A},x_{k}=1|\theta_{i}\right)dh_{k}^{A}+\int_{0}^{t_{h}}f\left(h_{k}^{A},x_{k}=-1|\theta_{i}\right)dh_{k}^{A}\right]\\ \;\;\;\;\;\;\;\;+{\left(\log\frac{1-P_{d}}{1-P_{f}}\right)}^{2}\left[\int_{t_{h}}^{+\infty }f\left(h_{k}^{A},x_{k}=-1|\theta_{i}\right)dh_{k}^{A}+\int_{0}^{t_{h}}f\left(h_{k}^{A},x_{k}=1|\theta_{i}\right)dh_{k}^{A}\right]\\ =\left[{\left(\log\frac{P_{d}}{P_{f}}\right)}^{2}p\left(b_{k}=1|\theta_{i}\right)+{\left(\log\frac{1-P_{d}}{1-P_{f}}\right)}^{2}p\left(b_{k}=0|\theta_{i}\right)\right]\left(\lambda_{1}+\lambda_{2}\right) \end{array}$$

## Appendix E. Calculation of the Derivative of $D(t_{3},t_{2},t_{1})$ to α

Rewrite $D(t_{3},t_{2},t_{1})$ as a function of *α*

(E1) $$D\left(\alpha \right)=m\left(t_{1}\left(\alpha \right)\right)-n\left(t_{3}\left(\alpha \right),t_{2}\left(\alpha \right)\right)$$

whose derivative is given by

(E2) $$\delta_{D}\left(\alpha \right)=\frac{dD(\alpha )}{d\alpha }=\frac{dD\left(\alpha \right)/dt_{1}}{d\alpha /dt_{1}}$$

Moreover, we can calculate

(E3) $$\begin{array}{cc} dD\left(\alpha \right)/dt_{1} & =d\left[t_{1}exp\left(-t_{1}^{2}\right)+\int_{t_{1}}^{\infty }exp\left(-h^{2}\right)dh-t_{3}exp\left(-t_{3}^{2}\right)+t_{2}exp\left(-t_{2}^{2}\right)-\int_{t_{3}}^{t_{2}}exp\left(-h^{2}\right)dh\right]/dt_{1}\\ & =-2t_{1}^{2}exp\left(-t_{1}^{2}\right)+2t_{3}^{2}exp\left(-t_{3}^{2}\right)\frac{dt_{3}}{dt_{1}}-2t_{2}^{2}exp\left(-t_{2}^{2}\right)\frac{dt_{2}}{dt_{1}} \end{array}$$

(E4) $$\begin{array}{cc} d\alpha /dt_{1} & =d\left[exp\left(-t_{1}^{2}\right)+exp\left(-t_{3}^{2}\right)-exp\left(-t_{2}^{2}\right)\right]/dt_{1}\\ & =-2t_{1}exp\left(-t_{1}^{2}\right)-2t_{3}exp\left(-t_{3}^{2}\right)\frac{dt_{3}}{dt_{1}}+2t_{2}exp\left(-t_{2}^{2}\right)\frac{dt_{2}}{dt_{1}} \end{array}$$

Specially, for the case $t_{3}=0$, we obtain

(E5) $$\begin{array}{c} dD\left(\alpha \right)/dt_{1}=\;\;\;-2t_{1}^{2}exp\left(-t_{1}^{2}\right)-2t_{2}^{2}exp\left(-t_{2}^{2}\right)\frac{dt_{2}}{dt_{1}}\\ d\alpha /dt_{1}=-2t_{1}exp\left(-t_{1}^{2}\right)+2t_{2}exp\left(-t_{2}^{2}\right)\frac{dt_{2}}{dt_{1}} \end{array}$$

And because $\lambda_{1}=\lambda_{2}$ has to be satisfied, the following equation is achieved

(E6) $$\begin{array}{c} dexp\left(-t_{1}^{2}\right)/dt_{1}=d\left(1-exp\left(-t_{2}^{2}\right)\right)/dt_{1}\\ \;\Rightarrow -2t_{1}exp\left(-t_{1}^{2}\right)=2t_{2}exp\left(-t_{2}^{2}\right)\frac{dt_{2}}{dt_{1}}\\ \Rightarrow \frac{dt_{2}}{dt_{1}}=-\frac{t_{1}exp\left(-t_{1}^{2}\right)}{t_{2}exp\left(-t_{2}^{2}\right)} \end{array}$$

Substituting Equation (E6) into Equation (E5) yields

(E7) $$\delta_{D}\left(\alpha \right)=\frac{t_{1}-t_{2}}{2}$$

Otherwise, for the case $t_{1}=t_{2}\;\;\;\;\left(\text{i.e.},\;\;\;t_{3}\geq 0\right)$, we have

(E8) $$\begin{array}{c} dD\left(\alpha \right)/dt_{1}=\;\;-4t_{1}^{2}exp\left(-t_{1}^{2}\right)+2t_{3}^{2}exp\left(-t_{3}^{2}\right)\frac{dt_{3}}{dt_{1}}\\ d\alpha /dt_{1}=-2t_{3}exp\left(-t_{3}^{2}\right)\frac{dt_{3}}{dt_{1}} \end{array}$$

Also due to $\lambda_{1}=\lambda_{2}$, it can be achieved

(E9) $$\begin{array}{c} dexp\left(-t_{1}^{2}\right)/dt_{1}=d\left(\exp\left(-t_{3}^{2}\right)-exp\left(-t_{1}^{2}\right)\right)/dt_{1}\\ \;\Rightarrow -4t_{1}exp\left(-t_{1}^{2}\right)=-2t_{3}exp\left(-t_{3}^{2}\right)\frac{dt_{3}}{dt_{1}}\\ \Rightarrow \frac{dt_{3}}{dt_{1}}=\frac{2t_{1}exp\left(-t_{1}^{2}\right)}{t_{3}exp\left(-t_{3}^{2}\right)} \end{array}$$

Further, $\delta_{D}\left(\alpha \right)=t_{1}-t_{3}$ is given.

## Appendix F. Proof of *D* (*λ_U_*) = *D* (*λ_L_* )

Beginning with $\left(P_{d}-P_{f}\right)=\left(P_{0d}-P_{m}\right)$, we get

(F1) $$\begin{array}{c} D\left(\lambda_{U}\right)=\int_{\log(\lambda_{U}q_{0}/q_{1})}^{+\infty }\left[f\left(\Lambda_{k}^{L}|\theta_{1}\right)-f\left(\Lambda_{k}^{L}|\theta_{0}\right)\right]d\Lambda_{k}\\ \;\;\;\;\;\;\;\;\;\;\;\;\;\;\;\;\;\;\;\;\;\;\;\;\;\;\;\;\;\;\;=-\int_{-\infty }^{\log(\lambda_{L}q_{0}/q_{1})}\left[f\left(\Lambda_{k}^{L}|\theta_{1}\right)-f\left(\Lambda_{k}^{L}|\theta_{0}\right)\right]d\Lambda_{k} \end{array}$$

From the total probability theory, we have

(F2) $$\int_{-\infty }^{+\infty }\left[f\left(\Lambda_{k}^{L}|\theta_{1}\right)-f\left(\Lambda_{k}^{L}|\theta_{0}\right)\right]d\Lambda_{k}=0$$

Then $D(\lambda_{U})$ can be rewritten as

(F3) $$\begin{array}{c} D\left(\lambda_{U}\right)=-\int_{-\infty }^{\log(\lambda_{L}q_{0}/q_{1})}\left[f\left(\Lambda_{k}^{L}|\theta_{1}\right)-f\left(\Lambda_{k}^{L}|\theta_{0}\right)\right]d\Lambda_{k}\\ \;\;\;\;\;\;\;\;\;\;\;\;\;\;\;\;\;\;\;\;\;\;\;\;\;\;\;\;\;\;=-(-\int_{\log(\lambda_{L}q_{0}/q_{1})}^{+\infty }\left[f\left(\Lambda_{k}^{L}|\theta_{1}\right)-f\left(\Lambda_{k}^{L}|\theta_{0}\right)\right]d\Lambda_{k})\\ \;\;\;\;\;\;\;\;\;\;\;\;\;\;\;\;\;\;\;\;\;\;\;\;\;\;\;\;\;=D(\lambda_{L}) \end{array}$$
